# Activation of the adipocyte CREB/CRTC pathway in obesity

**DOI:** 10.1038/s42003-021-02735-5

**Published:** 2021-10-22

**Authors:** Young-Sil Yoon, Weiyi Liu, Sam Van de Velde, Shigenobu Matsumura, Ezra Wiater, Ling Huang, Marc Montminy

**Affiliations:** 1 Peptide Biology Laboratories, The Salk Institute for Biological Studies, La Jolla, CA 92037 USA; 2 Department of Clinical Nutrition, Osaka Prefecture University, Habikino, Habikino City, Osaka Japan; 3 The Razavi Newman Integrative Genomics and Bioinformatics Core, The Salk Institute for Biological Studies, La Jolla, CA 92037 USA

**Keywords:** Obesity, Transcriptional regulatory elements

## Abstract

Obesity is a major risk factor for the development of type II diabetes. Increases in adipose tissue mass trigger insulin resistance via the release of pro-inflammatory cytokines from adipocytes and macrophages. CREB and the CRTC coactivators have been found to promote insulin resistance in obesity, although the mechanism is unclear. Here we show that high fat diet feeding activates the CREB/CRTC pathway in adipocytes by decreasing the expression of SIK2, a Ser/Thr kinase that phosphorylates and inhibits CRTCs. SIK2 levels are regulated by the adipogenic factor C/EBPα, whose expression is reduced in obesity. Exposure to PPARγ agonist rescues C/EBPα expression and restores SIK2 levels. CRTC2/3 promote insulin resistance via induction of the chemokines CXCL1/2. Knockout of CRTC2/3 in adipocytes reduces CXCL1/2 expression and improves insulin sensitivity. As administration of CXCL1/2 reverses salutary effects of CRTC2/3 depletion, our results demonstrate the importance of the CREB/CRTC pathway in modulating adipose tissue function.

## Abstract (web summary)

Yoon et al show that a high fat diet in mice activates the CREB/CRTC pathway in adipocytes by decreasing the expression of Salt Inducible Kinase 2 (SIK2), a Ser/Thr kinase that otherwise phosphorylates and sequesters CREB coactivators in the cytoplasm. Their data demonstrates the importance of the CREB/CRTC pathway in mediating effects of obesity on adipocyte function.

## Introduction

Obesity is associated with inflammatory changes in white adipose tissue (WAT) that lead to systemic insulin resistance and type 2 diabetes^1^. Sustained low-grade inflammation in this setting impairs triglyceride and glucose metabolism. Following their migration to WAT depots in response to circulating cytokines and chemokines, neutrophils promote infiltration of macrophages^2,3^, which in turn release TNFα and other pro-inflammatory cytokines that enhance insulin resistance^4–6^, in part via the induction of NF-κB. Indeed, pro-inflammatory cytokines have also been found to disrupt catecholamine signaling in adipocytes by stimulating phosphodiesterase PDE3B activity and blocking PKA-induced lipolysis^7^.

The cAMP pathway promotes cellular gene expression via the PKA-mediated stimulatory phosphorylation of CREB^8–10^ and inhibitory phosphorylation of the salt inducible kinases (SIKs). SIKs are active under basal conditions, where they phosphorylate and sequester the cAMP Responsive Transcriptional Coactivators (CRTCs) in the cytoplasm through an association with 14-3-3 proteins^11^. SIK2 is the most highly expressed of the three SIK family members in adipose tissue; and its downregulation in adipocytes of obese individuals is thought to contribute to insulin resistance^12,13^. Supporting this notion, mice with a knockout of SIK2 are glucose intolerant and insulin resistant^14^.

Dephosphorylation of the CRTCs in response to cAMP stimulates their translocation to the nucleus, where they bind to CREB over relevant promoters. Although cAMP signaling is itself associated with reduced inflammation in adipose tissue^15,16^, the adipocyte CREB/CRTC pathway has paradoxically been found to enhance insulin resistance in obesity^17–19^. Indeed, the loss of cAMP/PKA signaling in obese adipose tissue^20^ would be expected to block CREB/CRTC activation.

Here we examine the mechanism by which HFD feeding stimulates CRTC2 and CRTC3 activities in adipose tissue, leading to the downstream induction of a subset of pro-inflammatory genes in conjunction with NF-κB. Our results demonstrate an unexpected link between CREB/CRTC and cytokine signaling pathways in modulating adipose tissue function.

## Results

### Disruption of cAMP/PKA signaling and adipogenic gene expression in obesity

**Fig. 1:**
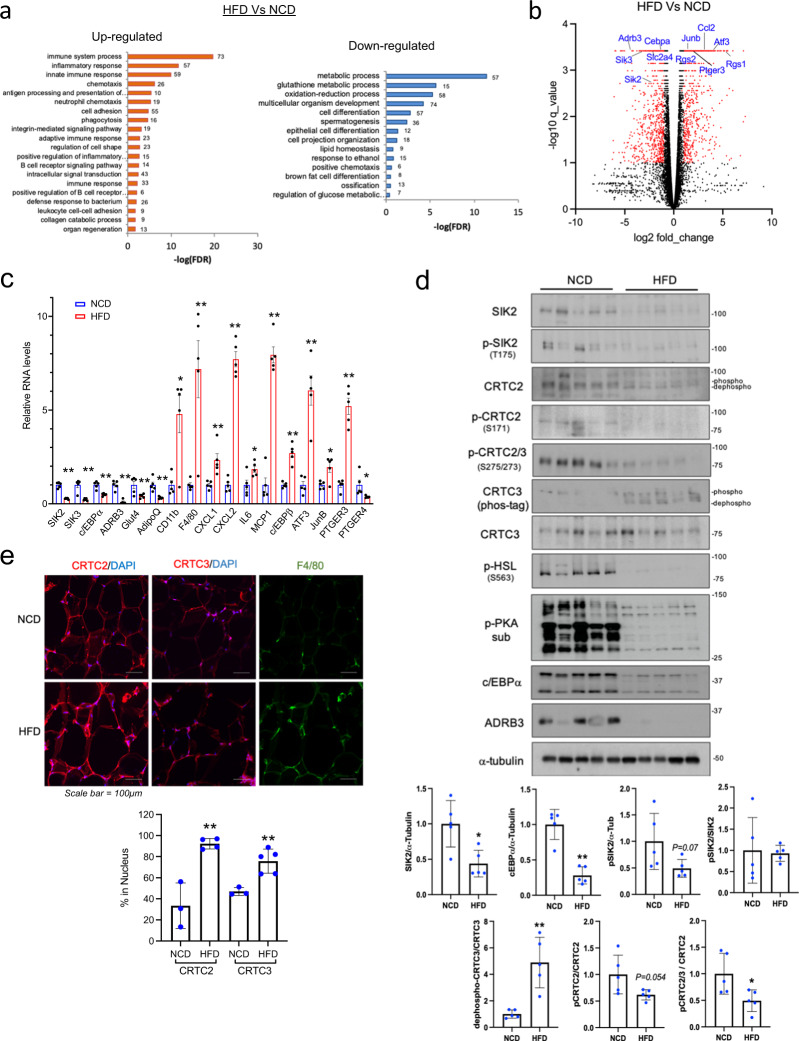
High fat diet feeding decreases SIK2 expression and activates CRTCs in adipose tissue. **a** RNA-seq analysis of genes expressed in eWAT from NCD and HFD fed (15 weeks) C57BL/6J mice. 1004 upregulated and 827 downregulated genes in HFD. Gene ontology (GO) of differentially regulated gene clusters shown. The numbers of genes for each GO term indicated. **b** Volcano plot showing relative effects of high-fat diet (HFD) versus normal chow diet (NCD) feeding (15 weeks) on age-matched C57BL/6J mice. Genes selected for greater than 2-fold change (log FC > 1) with a *q*-value (adjusted *P*-value) < 0.05 (*n* = 3 per group). **c** q-PCR analysis of gene expression in eWAT from age-matched NCD and HFD fed mice (15 weeks) (**P < 0.01, *P < 0.05, *t*-test; *n* = 5 per group). **d** Western blot analysis of eWAT from age-matched NCD-fed and HFD-fed mice (15 weeks). Effect of HFD on SIK2 protein amounts and CRTC2/3 phosphorylation shown. Relative protein amounts for metabolic (C/EBPα and ADRB3) genes under NCD or HFD conditions indicated. PKA activity in eWAT evaluated by blotting for phospho-HSL and phospho-PKA substrates. Each lane represents one animal. Densitometry analysis was performed using Image J software. Data from experiments repeated more than three times. **e** Immunofluorescence images showing the effect of 6 weeks HFD on CRTC2/3 nuclear staining and macrophage (F4/80) infiltration in eWAT. Bottom, quantification of cells in which CRTC2/3 expression colocalizes with nuclear DAPI (**P < 0.01, *t*-test; *n* = 3–5 per group).

We evaluated the effects of obesity on gene expression in WAT by feeding C57BL/6J mice a 60% HFD for 15 weeks (Fig. 1a, b). Body weight and fasting blood glucose levels were elevated in HFD-fed relative to NCD-fed mice (Supplementary Fig. 1a). Gene ontology analysis of RNA-seq data from epidydimal WAT (eWAT) mRNA, revealed upregulation of “inflammatory response” genes and downregulation of “glucose and lipid metabolism” genes in response to HFD (Fig. 1a). Metabolic genes with reduced expression under HFD conditions include the GLUT4 glucose transporter (SLC2A4), the Beta 3 Adrenergic receptor (ADRB3), salt inducible kinases (SIK2, SIK3) as well as CAAT/enhancer-binding protein alpha (C/EBPα). The expression of these HFD-downregulated genes is inversely correlated with insulin resistance and obesity in mice and humans^12,21–24^.

In keeping with the induction of pro-inflammatory genes in response to HFD^1,25,26^, NF-κB target genes such as c–c motif chemokine ligand 2 (CCL2; also referred to as MCP1), IFN responsive regulator of G protein signaling 1 (RGS1), and prostaglandin E2 receptor 3 (PTGER3; also referred to as EP3) were all upregulated in response to HFD feeding (Fig. 1b, c).

Adipose tissue contains a number of cell types, including immune and endothelial cells as well as pre-adipocytes and mature adipocytes. To identify specific subsets of cells that display similar profiles of metabolic and inflammatory gene expression as adipose tissue in response to HFD, we performed fluorescence-activated cell sorting (FACS) studies. Immune and endothelial cells from eWAT showed little change in either inflammatory or metabolic gene expression under HFD vs. NCD conditions; but pre-adipocytes and mature adipocytes showed profiles of both up- and downregulated genes that mimicked those of intact adipose tissue (Supplementary Fig. 1e, f).

HFD feeding has been found to block cAMP signaling in adipocytes through the IκB Kinase (IKK)-mediated phosphorylation and activation of the phosphodiesterase PDE3B^7^. In line with this observation, PKA activity was decreased in HFD adipose tissue of WT mice, by western blot assay of both eWAT and iWAT using phospho-PKA substrate antibody as well as antisera against PKA-phosphorylated Hormone Sensitive Lipase (HSL) and phospho-AMPK^27^ (Fig. 1d and Supplementary Fig. 1b, c).

In addition to its inhibitory effects on the cAMP pathway, HFD feeding reduced insulin signaling (P-AKT, pSer-IRS1), glucose transporter (GLUT4), and β-3 adrenergic receptor (ADRB3) protein amounts in HFD eWAT (Fig. 1d and Supplementary Fig. 1b). Moreover, mRNA and protein levels for the Ser/Thr kinase SIK2 are also downregulated in adipose tissue from HFD fed relative to NCD-fed mice (Fig. 1b–d and Supplementary Fig. 1c).

### Effect of obesity on SIK2 expression in adipose tissue

The Ser/Thr kinase SIK2 is the most highly expressed of the three SIK family members in adipocytes, where it promotes triglyceride homeostasis and whole-body insulin sensitivity^12,14,28–30^. SIK activity is dependent on phosphorylation (at Thr175) by LKB1, a master kinase for AMPK family members. Triggering of the cAMP pathway blocks SIK2 activity via PKA-mediated phosphorylation^31^. Recognizing that SIK2 inhibits CREB/CRTC signaling, under basal conditions, by phosphorylating CRTCs at 14-3-3 binding sites^11^, we evaluated the phosphorylation status of these proteins in adipose tissue.

Consistent with the decrease in total as well as phospho (Thr175)-specific^28^ SIK2 protein amounts (Fig. 1d), CRTC2 and CRTC3 are dephosphorylated to a greater extent in eWAT as well as iWAT from HFD-fed compared to NCD-fed mice (Fig. 1d and Supplementary Fig. 1c). As a result, CRTC2/3 nuclear staining is increased in eWAT from HFD-fed mice, by immunohistochemical analysis (Fig. 1e and Supplementary Fig. 1d). These results indicate that the loss of SIK2 expression in obese WAT is sufficient to promote increases in nuclear CRTC2/3.

**Fig. 2:**
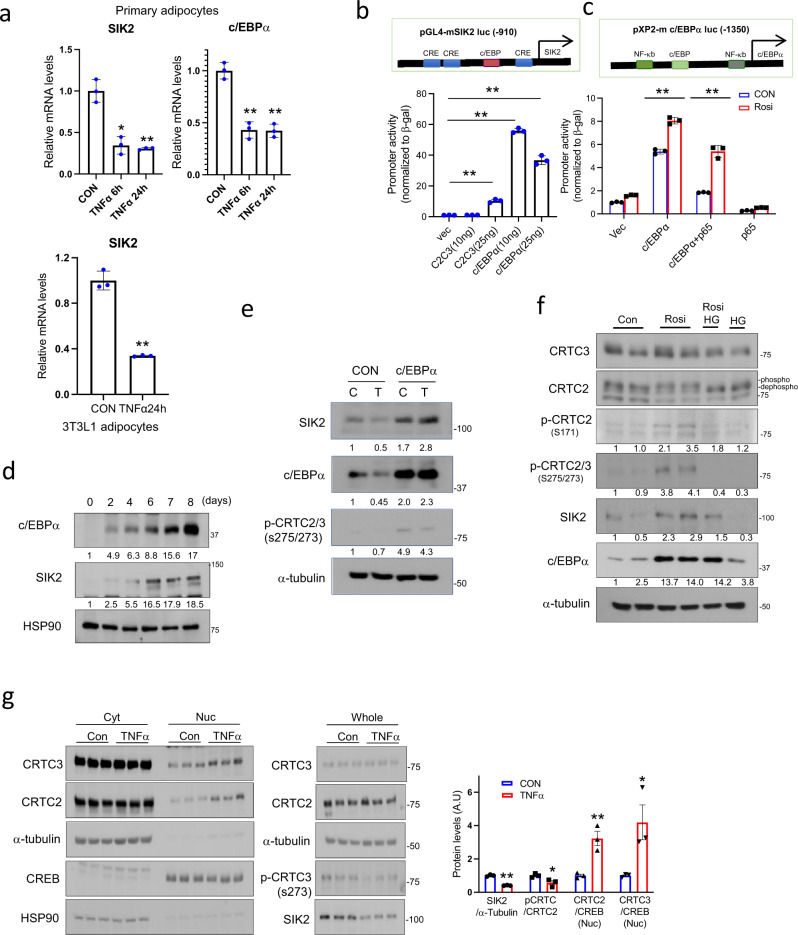
TNFα activates CRTCs by decreasing c/EBPα and SIK2 expression in adipocytes. **a** Effect of TNFα on SIK2 and C/EBPα mRNA amounts in differentiated primary white adipocytes and 3T3-L1 adipocytes. TNFα (10 ng/ml) treatment for indicated times (**P < 0.01, *P < 0.05, one-way ANOVA, *t*-test; *n* = 3 per group). Data from experiments repeated three or more times. **b** Relative effect of CRTC2/3 (C2C3) and C/EBPα overexpression on mouse SIK2 promoter activity in HIB1b cells shown. Luciferase activity normalized to RSV-β gal activity (**P < 0.01, *t*-test; *n* = 3 per group). **c** Luciferase assay showing the effect of c/EBPα and NF-κB p65 overexpression on c/EBPα promoter activity in HIB1b cells. Luciferase activity normalized to RSV-β gal activity (**P < 0.01, one-way ANOVA; *n* = 3 per group). Treatment with PPARγ agonist Rosiglitazone (Rosi;1 µM, 16 h) indicated. **d** Immunoblot showing relative time course for SIK2 and c/EBPα expression in differentiating 3T3-L1 cells. **e** Immunoblots showing effects of C/EBPα overexpression on endogenous SIK2 protein levels in HIB1b cells exposed to TNFα. Cells were incubated with lentiviral C/EBPα or control vector for 2 days. Infected cells were then exposed to TNFα (T, 10 ng/ml) for 6 h. Densitometry analysis of SIK2 expression and CRTC phosphorylation shown. **f** Effect of PPARγ agonist (Rosi) on endogenous SIK2 protein amounts and CRTC phosphorylation in 3T3-L1 cells. Undifferentiated cells exposed to Rosi (5 µg/ml) for 24 h and HG9-91-01 (HG;10 µM) for the final 1 h. Immunoblots show C/EBPα and SIK2 protein amounts as well as phosphorylated forms of CRTC2/3. **g** Effect of TNFα (10 ng/ml, 6 h) on CRTC nuclear translocation in 3T3-L1 adipocytes. Immunoblots show CRTC2/3 protein amounts in nuclear and cytoplasmic fractions. p-CRTC and SIK2 amounts in whole-cell lysates indicated. Each lane represents one plate of cells. Right, bar graph showing densitometry analysis of fractionation study (**P < 0.01, *t*-test; *n* = 3 per group). Numbers below each lane in **d**–**f** indicate relative expression levels. Data in **a**–**c** represent mean ± SD and data in **g** represent mean ± SEM.

Obesity triggers the expression and release of the pro-inflammatory cytokine TNFα from adipocytes and resident macrophages in adipose tissue, where it interferes with insulin signaling and triglyceride metabolism^4,32^. We explored the potential role of this cytokine in modulating SIK2 expression. Exposure of primary adipocytes or 3T3-L1 adipocytes to TNFα (6 h) decreased mRNA amounts for both SIK2 and C/EBPα (Fig. 2a).

Within the SIK2 promoter, we noted consensus binding sites for C/EBPα and CREB. Overexpression of CRTC2/3 had only modest effects on SIK2 promoter activity in transient assays using a SIK2 luciferase reporter, but C/EBPα overexpression strongly upregulated this reporter in HIB1b cells (Fig. 2b). In keeping with the ability for C/EBPα to auto-regulate its own promoter, C/EBPα overexpression also potentiated the activity of a C/EBPα reporter construct (Fig. 2c); these effects were further enhanced by co-treatment with the PPARγ agonist Rosiglitazone (Rosi). By contrast, overexpression of p65, the trans-activating subunit of the NF-κB hetero-dimeric complex^33^, inhibited C/EBPα reporter activity in HIB1b cells (Fig. 2c).

In keeping with the effects of C/EBPα on SIK2 reporter activity, exposure of 3T3-L1 cells to differentiation medium increased endogenous C/EBPα protein amounts, followed by increases in SIK2 expression (Fig. 2d). Modest C/EBPα overexpression in HIB1b cells rescued TNFα-induced decreases in SIK2 expression. As a result, phosphorylation of endogenous CRTC2/3 increased in cells over-expressing C/EBPα (Fig. 2e).

PPARγ has been shown to promote adipogenesis by stimulating C/EBPα expression and to inhibit inflammation in WAT by suppressing NF-κB activity^34,35^. Exposure of 3T3-L1 cells to Rosi upregulated endogenous levels of C/EBPα, leading to increases in SIK2 protein amounts, and to the subsequent inhibitory phosphorylation of CRTC2 and CRTC3 (Fig. 2f). Consistent with a requirement for SIK2, exposure to SIK inhibitor HG9-91-01(HG) blocked the effects of Rosi on CRTC2/3 phosphorylation. Conversely, treatment with TNFα reduced SIK2 levels, triggering CRTC2/3 dephosphorylation and nuclear translocation as revealed in subcellular fractionation studies of 3T3-L1 adipocytes (Fig. 2g). Taken together, these results suggest that PPARγ and TNFα modulate the CREB/CRTC pathway in adipocytes by enhancing or inhibiting SIK2 expression.

### Cooperative effects of CRTCs and NF-κB on CXCL1 expression

Having seen that HFD or TNFα treatment increases the nuclear accumulation of CRTC2/3 in adipocytes, we searched for downstream CREB target genes that contribute to the inflammatory and metabolic changes associated with obesity. Exposure of brown pre-adipocytes to Forskolin (FSK) stimulated the expression of 20 genes 8-fold or better (log FC ≥ 3); many of these contain CREB binding sites in their promoters (Supplementary Fig. 2 and Supplementary Table 1). Among these candidate targets, we noticed that the inflammatory chemokine (C-X-C motif) ligand 1 (CXCL1) gene was upregulated in response to FSK.

CXCL1 and its paralog CXCL2 have been shown to mediate neutrophil recruitment and activation to different tissues in response to infection. Mouse CXCL1 and CXCL2 (CXCL1/2) are well-established functional homologs of human CXCL8 (IL8), whose expression is also induced by NF-κB^36–38^ in obesity and insulin resistance^39–44^.

**Fig. 3:**
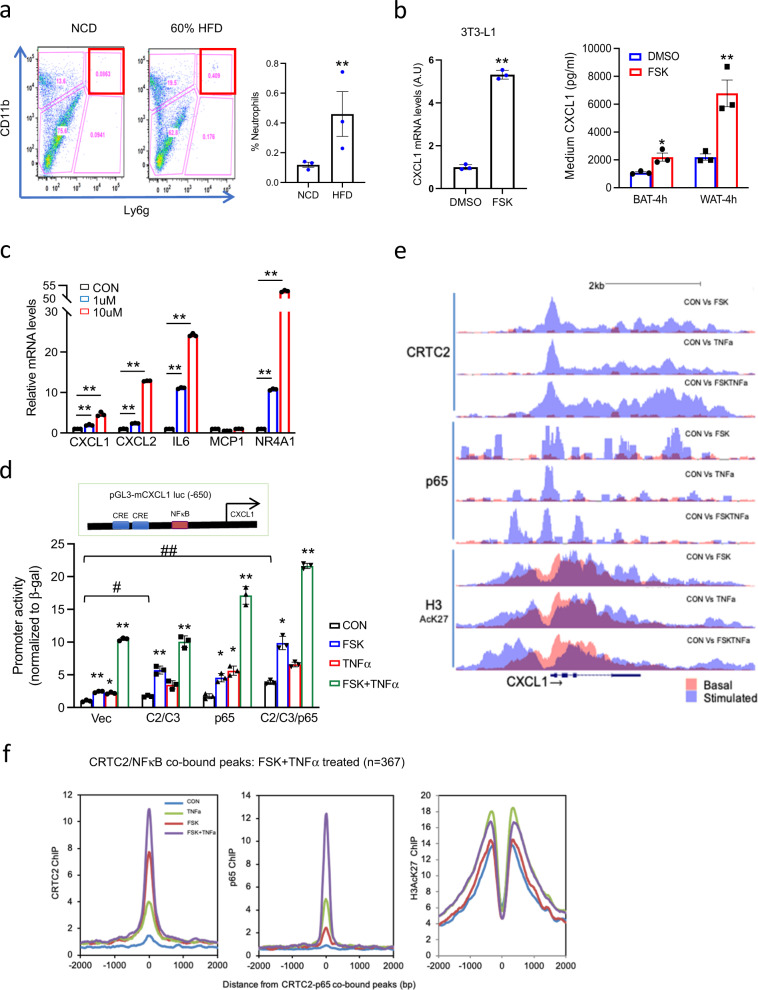
Cooperative effects of CRTC and NF-κB on a subset of cytokine genes in adipocytes. **a** Flow cytometry analysis of neutrophil populations (CD11b^+^; Ly6G^+^) in WAT from mice fed NCD or HFD for 6 weeks (**P < 0.01, *t*-test; *n* = 3 per group). Right, bar graph showing the relative abundance of neutrophils in eWAT from NCD and HFD fed mice. **b** Left, the effect of FSK (10 µM, 1 h) on CXCL1 expression in 3T3-L1 adipocytes. Right, the effect of FSK on the release of CXCL1 from primary adipocytes. Stromal vascular fraction (SVF) cells from eWAT or BAT were incubated in a differentiation cocktail for 7 days and then exposed to FSK (10 µM) for 4 h. Release of CXCL1 into the medium shown (**P < 0.01, *P < 0.05, *t*-test; *n* = 3 each). **c** Effect of SIK inhibitor HG9-91-01 (1 µM or 10 µM, 3 h) on endogenous mRNA amounts for CXCL1, CXCL2, and other genes in differentiated 3T3-L1 cells (**P < 0.01, *P < 0.05, one-way ANOVA; *n* = 3 per each). **d** Transient assay of HIB1b cells showing the effect of CRTC2/3 and p65 overexpression on mouse CXCL1 promoter activity. Cells were exposed to FSK (10 µM) and TNFα (10 ng/ml) for 4 h (**P < 0.01, *P < 0.05, one-way ANOVA; *n* = 3 per each). **e** ChIP-seq analysis of CRTC2, NF-κB p65, and H3AcK27 occupancy over the mouse CXCL1 promoter in 4-day differentiated 3T3-L1 cells. Treatment with FSK (10 µM), TNFα (10 ng/ml) alone and together for 1 h as indicated. Orange, basal occupancy; light blue, occupancy following stimulation with FSK and TNFα; dark blue, the overlap between basal and FSK/TNFα inducible binding. **f** Histograms showing the effect of TNFα and FSK alone and together on CRTC2, NF-κB p65, and H3AcK27 occupancy over CRTC2-NFκB p65-co-bound loci in 4d differentiated 3T3-L1 adipocytes (*n* = 367). Data in **a**, **b** represent the mean ± SEM, and data in **c**, **d** represent the mean ± SD.

HFD feeding stimulated the expression and release of CXCL1/2 from adipose tissue into the circulation (Fig. 1c and Supplementary Fig. 3a), leading to increases in neutrophil migration and infiltration of eWAT (Fig. 3a). Consistent with this scenario, exposure of 3T3-L1 adipocytes or primary adipocytes to FSK also induces CXCL1 expression (Fig. 3b). Indeed, exposure of 3T3-L1 adipocytes to a more physiologic cAMP stimulus (β3 adrenergic receptor agonist; CL316,243) also augments CXCL1 expression, albeit more modestly than FSK (Supplementary Fig. 3b).

In keeping with the role of SIK2 in suppressing CRTC activity, exposure to SIK inhibitor HG upregulated CXCL1/2 expression comparably to FSK (Fig. 3c). SIK inhibition also increased the expression of other inflammatory genes (IL6) that have CREB and NF-κB binding sites on their promoters but not genes like MCP1, an NF-κB target lacking a CREB binding site.

Recognizing that the CXCL1 promoter contains two cAMP response elements (CREs) as well as an NF-κB binding site, we evaluated the extent to which these regulatory pathways modulate the expression of this gene. Exposure to either FSK or TNFα increased CXCL1 reporter activity 2–3 fold, while exposure to both FSK and TNFα increased reporter activity synergistically (10-fold) (Fig. 3d and Supplementary Fig. 3c). We also observed cooperativity between NF-κB and CREB/CRTC pathways on a human CXCL8 (IL8) reporter in transient assays of HEK293T cells (Supplementary Fig. 3d). Indeed, co-expression of CRTC2/3 and p65 potentiated effects of FSK and TNFα on IL8 reporter activity. These results indicate that NF-κB and CREB/CRTC pathways co-regulate the expression of CXCL1/2 and perhaps other genes in adipocytes.

TNFα and other cytokines stimulate pro-inflammatory gene expression in part through induction of the inhibitor of nuclear factor κB kinase (IKK), which phosphorylates IκBs and thereby promotes their degradation^45,46^. Exposure to IKK inhibitor (IKK16) blocked the induction of the CXCL1 and CXCL2 genes in response to TNFα. Remarkably, IKK16 also interfered with induction of the CXCL1 and CXCL2 genes by SIK inhibitor (HG), suggesting that IKK activity is necessary for the cooperativity between NFκB and CREB pathways (Supplementary Fig. 3e).

We wondered whether FSK and TNFα exert distinct or overlapping effects on p65 and CRTC2 recruitment to the CXCL1 promoter. In chromatin-immunoprecipitation sequencing (ChIP-seq) studies of differentiated 3T3-L1 adipocytes (Fig. 3e and Supplementary Fig. 4), CRTC2 occupancy over the CXCL1 promoter is low under basal conditions but increases following exposure (1 h) to FSK. In line with its ability to stimulate CRTC2/3 nuclear translocation, treatment with TNFα also promotes CRTC2 recruitment to the CXCL1 promoter (Fig. 3e). Moreover, exposure to both FSK and TNFα further enhances CRTC2 as well as p65 occupancy over their respective binding sites. CRTC2 occupancy extends through the CXCL1 gene body, suggesting that it may contribute to both the transcriptional initiation and productive elongation of RNA polymerase II complexes along this gene.

In addition to their effects on CXCL1, FSK and TNFα increased the occupancy of both CRTC2 and p65 at 367 co-bound loci (Fig. 3f). 70 of these loci have CRE(s) (TGACG), and 86 have NFκB motif(s) (GGAAWTTCCC), while 12 have both consensus CRE and NFκB binding sites. Some co-bound promoters (e.g., CCL2, CXCL5) lack consensus CREs, but they contain Jun/AP1 binding sites, which also appear capable of mediating recruitment of CRTCs to the promoter, potentially via an association with jun/fos family members^47^ (Supplementary Fig. 4a–c). Consistent with this notion, CCL2 and CXCL5 promoters lack consensus CREs, yet they are bound by CRTC2; and these genes are downregulated in dAKO adipocytes relative to wild-type adipocytes exposed to TNFα (Supplementary Fig. 4d).

### Knockout of CRTC2/3 improves adipose function in obesity

**Fig. 4:**
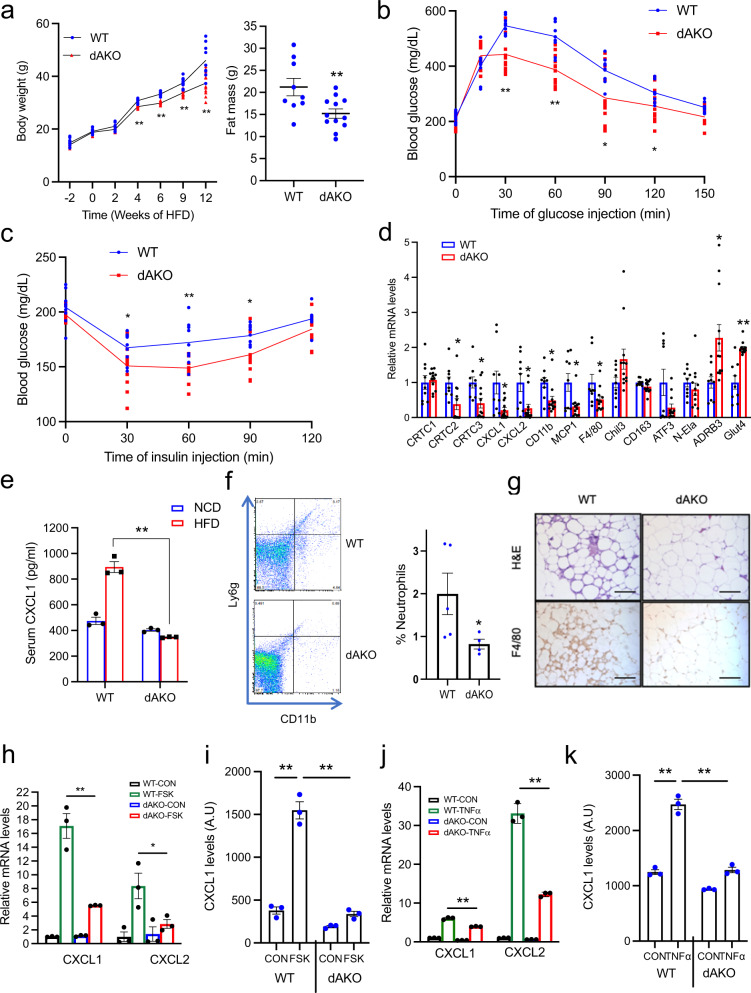
Mice with a knockout of both CRTC2 and CRTC3 in adipose tissue are resistant to HFD-induced obesity. **a** Body weight gain and fat mass in WT and double (CRTC2/3) adipose KO (dAKO) littermates after HFD feeding for 12 weeks (**P < 0.01, *t*-test; *n* = 9, 12 per group). HFD started at 4–5 weeks of age. **b**, **c** Glucose (1 g/kg glucose, IP) (**b**) and Insulin (1 U/kg insulin) tolerance testing (**c**) of dAKO and WT littermates. Mice were fed an HFD for 12 weeks (**P < 0.01, *P < 0.05, *t*-test; *n* = 9, 12 per group). **d** Q-PCR analysis of mRNA amounts for immune-related and metabolic genes in adipose from dAKO and WT littermates under HFD conditions for 12 weeks (*P < 0.05, *t*-test; *n* = 9, 12 per group). **e** Circulating concentrations of CXCL1 in dAKO mice and WT littermates after 6 weeks HFD or NCD feeding (**P < 0.01, *t*-test; *n* = 3 per group). **f** Flow cytometry analysis showing neutrophil (CD11b^+^; Ly6g^+^) numbers in eWAT from dAKO and WT littermates after 6 weeks HFD (*P < 0.05, *t*-test; *n* = 5, 4 per group). **g** H&E and macrophage (F4/80) staining in eWAT tissue sections from WT and dAKO mice after HFD feeding for 12 weeks. Representative images shown from more than 3 mice per group. **h** Relative mRNA amounts for CXCL1 and CXCL2 in differentiated primary adipocytes from dAKO or WT mice. Exposure to FSK (10 µM, 2 h) indicated. **i** Effect of FSK (10 µM, 6 h) on the secretion of CXCL1 from differentiated primary WT or dAKO adipocytes. **j** Effect of TNFα (10 ng/ml, 2 h) on the expression of CXCL1 and CXCL2 genes in differentiated primary adipocytes from dAKO or WT mice. **k** Effects of TNFα (10 ng/ml, 12 h) on the secretion of CXCL1 from differentiated primary adipocytes of dAKO and WT littermates. Significance determined by one-way ANOVA for **h**–**k** (**P < 0.01, *<0.05, *n* = 3 per group). Data in **a**–**f** and **i**, **k** represent the mean ± SEM, and **h**, **j** represent the mean ± SD.

Of the three CRTC family members, CRTC2 and CRTC3 are expressed at the highest levels in adipose tissue relative to CRTC1, which is preferentially expressed in the brain. To determine the role of CRTCs in adipose tissue, we crossed mice with floxed alleles of both CRTC2/3 with Adipoq-Cre transgenic mice expressing Cre recombinase under the control of the adipose-specific adiponectin promoter. CRTC2/3 expression in WAT is reduced by more than 70% in CRTC2/3 double knockout (dAKO) mice by qPCR and western blotting analysis (Fig. 4d and Supplementary Fig. 5a, b). Based on the proposed effects of CRTC2/3 on CXCL1/2 expression and insulin resistance, we evaluated whether depletion of CRTC2/3 in adipose tissue modulates the effects of HFD on insulin signaling and glucose metabolism.

Although they were indistinguishable from WT littermates under NCD conditions, dAKO mice gained less weight and had a lower fat mass in the context of HFD feeding, beginning after 4 weeks of HFD (Fig. 4a and Supplementary Fig. 5c). HFD-fed dAKO mice have lower fasting blood glucose levels; glucose and insulin tolerance is also improved in dAKO relative to WT (Fig. 4b, c and Supplementary Fig. 5d).

We performed indirect calorimetry studies to further characterize energy metabolism in dAKO mice (Supplementary Fig. 5e). Consistent with their lower body weight on HFD, dAKO mice have elevated oxygen consumption rate and energy expenditure relative to WT littermates. Despite these differences, physical activity and food intake appear comparable between CRTC2/3 mutant and WT littermates.

In keeping with the effects of CRTC2/3 on CREB target gene expression in adipose tissue, mRNA amounts for CXCL1 and CXCL2 in WAT are downregulated in dAKO mice relative to WT (Fig. 4d). Correspondingly, neutrophil numbers in adipose tissue from HFD-fed dAKO mice are 2-fold lower than in HFD-fed WT adipose tissue. mRNA amounts for neutrophil (CD11b) and M1 macrophage (F4/80) markers are decreased in dAKO WAT, suggesting that a reduction in CXCL1/2 mediated neutrophil recruitment also attenuates adipose tissue infiltration by other inflammatory cells.

In line with the improvements in adipose tissue inflammation, cAMP and insulin signaling pathways were more active in dAKO mice relative to wild-type (Supplementary Fig. 5f, g). Metabolic (ADRB3, GLUT4) gene expression was also increased in eWAT from dAKO mice relative to WT (Fig. 4d). c/EBPα protein amounts are elevated in dAKO eWAT, and SIK2 expression is correspondingly higher.

HFD feeding increases circulating levels of CXCL1 in WT mice, but CXCL1 expression remains low in HFD-fed dAKO mice (Fig. 4e). In line with the loss of CRTC2/3 expression, dAKO mice have lower numbers of HFD-inducible neutrophils (CD11b^+^; Ly6g^+^) and macrophages (F4/80^+^) in eWAT (Fig. 4f, g). Consistent with these metabolic improvements, dAKO adipocytes expressed and secreted lower levels of CXCL1/2 following exposure to FSK or TNFα compared to wild-type cells (Fig. 4h–k).

### CXCL1/2 mediate effects of CRTC1/2 on HFD-induced inflammation

Based on the effects of HFD in promoting cytokine gene expression, we wondered whether CRTC2/3 depletion would attenuate the reactivity of WAT tissue to an inflammatory stimulus. Intra-peritoneal administration of low-dose lipopolysaccharide (LPS; 500 ng/kg, 3 h) stimulated the expression of inflammatory genes that are targets of both CRTC and NFκB (CXCL1, CXCL2, IL6) or NFκB alone (TNFα, IL1β, MCP1) in WT eWAT. Depletion of CRTC2/3 reduced expression of NFκB/ CRTC coregulated targets and to a lesser extent NFκB selective targets in dAKO eWAT (Supplementary Fig. 6). Collectively, these studies suggest that CRTC2/3 acts upstream of NFκB to promote the expression of cytokine genes in adipose tissue.

**Fig. 5:**
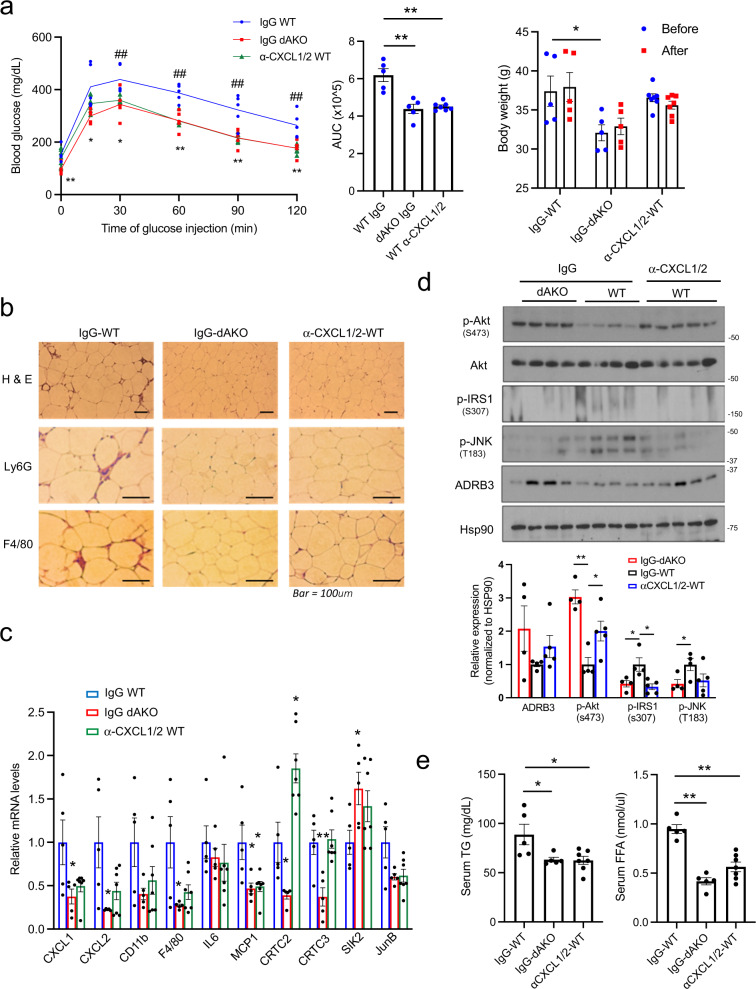
Immuno-depletion of CXCL1/2 protects against HFD-induced immune cell infiltration into adipose tissue. **a** Glucose tolerance testing (1 g/kg glucose) of dAKO and WT mice injected with neutralizing antibodies against CXCL1/2 (α-CXCL1/2) or IgG control (** or ^##^*P* < 0.01, *P < 0.05, *t*-test; *n* = 5, 5, 7 per group). Mice were fed a HFD for 9 weeks. Middle, area under curve (AUC) showing effect of dAKO and CXCL1/2 immuno-neutralization (**P < 0.01, one-way ANOVA; *n* = 5, 5, 7 per group). Right, Changes in body weight before and after injection of control IgG or anti-CXCL1/2 antiserum (*P < 0.05, one-way ANOVA; *n* = 5, 5, 7 per group). **b** Microscope sections of eWAT from IgG or α-CXCL1/2 injected WT mice, versus IgG injected dAKO mice under HFD feeding conditions for 9 weeks. Sections were stained using antisera against neutrophil (Ly6G) or macrophage marker (F4/80). Representative images from 5 mice per group shown. **c** Expression of immune-related genes in eWAT from IgG injected WT, IgG injected dAKO, and α-CXCL1/2 injected WT HFD fed mice (**P < 0.01, *P < 0.05, one-way ANOVA; *n* = 5, 5, 7 per group). **d** Immunoblots of eWAT showing effect of α-CXCL1/2 immuno-neutralization in HFD-fed (9 weeks) WT mice on insulin and stress signaling. Bottom, densitometric analysis of immunoblots (**P < 0.01, *P < 0.05, one-way ANOVA; *n* = 5, 5, 7 per group). **e** Circulating Triglyceride (TG) and Free fatty acid (FFA) concentrations in serum from dAKO and α-CXCL1/2-neutralized mice (**P < 0.01, *P < 0.05, one-way ANOVA; *n* = 5, 5, 7 per group). Data in **a**, **c**–**e** represent the mean ± SEM.

Having seen that CXCL1/2 expression is reduced in dAKO mice, we evaluated the extent to which these chemokines contribute to the effects of HFD on inflammation and lipid metabolism. Administration of CXCL1/2 neutralizing antiserum during the HFD feeding regimen reduces circulating concentrations of CXCL1/2 proteins to a similar extent in WT mice as those in dAKO mice (Supplementary Fig. 7a). Neutrophil and macrophage infiltration into adipose tissue is downregulated by immunohistochemical analysis of eWAT sections from immuno-neutralized mice; and glucose tolerance is correspondingly improved (Fig. 5a, b). Indeed, neutrophil and macrophage marker gene expression (CD11b, F4/80, MCP1) is also reduced in eWAT from anti-CXCL1/2 immuno-neutralized mice compared with control (Fig. 5c). In RNA-seq studies of eWAT, most of the gene clusters that are upregulated in HFD versus NCD feeding correspond to immune response and chemokine signaling (Supplementary Fig. 7d). mRNA amounts for these genes are decreased in both dAKO and anti-CXCL1/2 antiserum injected mice.

Consistent with this decrease in pro-inflammatory gene expression, insulin signaling, evaluated by relative pSer(473)AKT and pSer(307)IRS1 protein amounts, is enhanced while stress signaling (pThr(183)JNK) is attenuated in eWAT from CXCL1/2-neutralized mice (Fig. 5d). As a result, circulating triglyceride and free fatty acid concentrations are decreased in HFD-fed dAKO and CXCL1/2 immuno-neutralized mice relative to WT. Indeed, lipid accumulation in the liver is reduced in dAKO mice (Fig. 5e and Supplementary Fig. 7b, c). Collectively, these results indicate that the induction of CXCL1/2 by CRTC2/3 in adipose tissue during HFD feeding, contributes in part to the development of insulin resistance.

### Effect of CXCL1 knockout on diet-induced insulin resistance

**Fig. 6:**
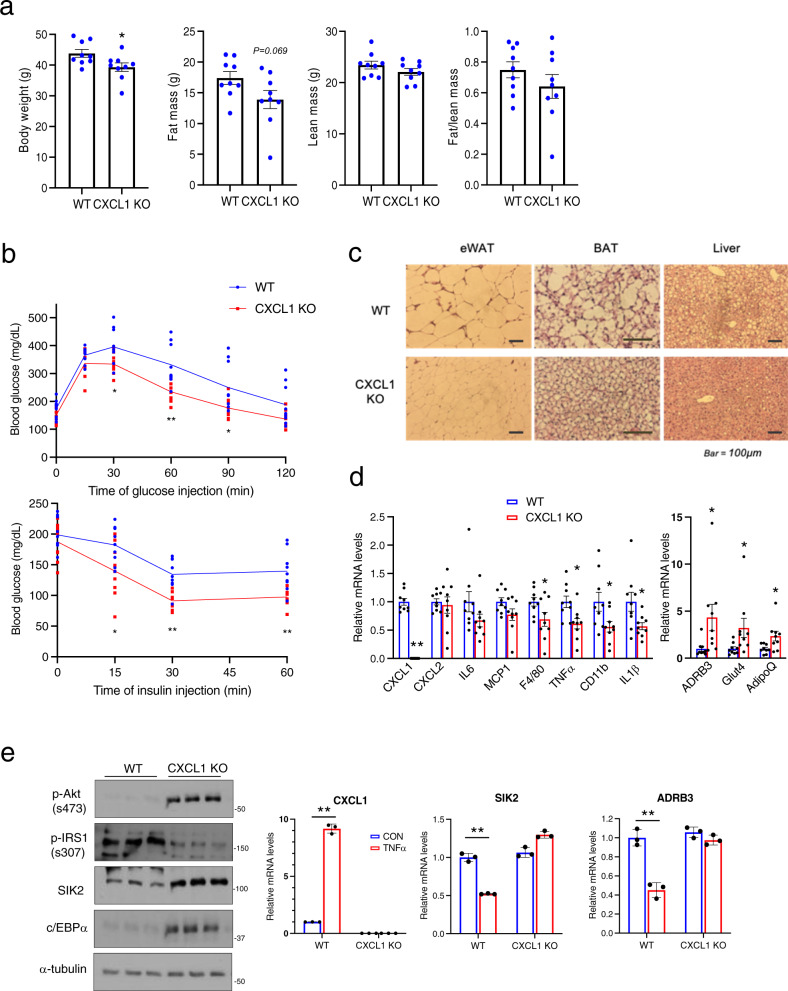
Knockout of CXCL1 corrects HFD-induced glucose intolerance and metabolic gene expression in WAT. **a** Body weight and fat mass in CXCL1 KO and WT littermates following HFD feeding for 10 weeks (*P < 0.05, *t*-test; *n* = 9, per group). **b** Glucose tolerance (top, 1 g/kg of glucose) and insulin tolerance (bottom, 1 U/kg of insulin) testing of CXCL1 KO and WT littermates (**P < 0.01, *P < 0.05, *t*-test; *n* = 9, per group). Mice were fed a HFD for 10 weeks. **c** H&E staining of sections from eWAT, BAT, and livers of CXCL1 KO mice and WT littermates. Representative images from more than 5 mice per group. **d** Relative mRNA amounts for immune-related and metabolic genes by quantitative PCR of eWAT from HFD fed (10 weeks) CXCL1 KO and WT littermates (**P < 0.01, *P < 0.05, *t*-test; *n* = 9 per group). **e** Effect of CXCL1 deficiency on insulin signaling in differentiated white adipocytes from CXCL1 KO and WT littermates. Right, qPCR analysis showing effect of CXCL1 depletion on TNFα (10 ng/ml, 6 h) mediated changes in SIK2 and c/EBPα (**P < 0.01, *P < 0.05, one-way ANOVA; *n* = 3 per group). Data in **a**, **b**, and **d** represent mean ± SEM, and data in **e** represent mean ± SD.

Based on the effects of CXCL1/2 immuno-neutralization on insulin signaling, we employed CXCL1 KO mice to evaluate whether this chemokine contributes to HFD-induced insulin resistance (Fig. 6). Under HFD conditions, CXCL1 KO mice had lower body weight and a tendency toward lower fat mass (*P* = 0.07, Fig. 6a). Glucose metabolism was improved in CXCL1 KO mice relative to WT littermates by intraperitoneal glucose (IPGTT) and insulin tolerance testing (ITT) (Fig. 6b and Supplementary Fig. 8a). Indeed, CXCL1 KO mice had lower circulating concentrations of triglyceride and free fatty acids relative to WT (Fig. 6c and Supplementary Fig. 8b, c). Consistent with an improvement in lipid metabolism, triglyceride stores in BAT and liver are reduced in HFD fed CXCL1 KO mice relative to WT (Fig. 6c).

Inflammatory gene expression (F4/80, TNFα, CD11b, IL1β) is also downregulated in CXCL1 KO adipose tissue, while metabolic gene expression (ADRB3, Glut4, AdipoQ) is elevated. These salutary effects are associated with increases in insulin signaling (p-AKT) and adipogenic (c/EBPα) gene expression (Fig. 6e). In keeping with the stimulatory effects of c/EBPα on the SIK2 gene, protein levels for SIK2 are also increased in eWAT from HFD-fed CXCL1 KO relative to WT mice. Collectively, these results demonstrate that CXCL1 contributes to the inflammatory and metabolic changes that accompany HFD feeding (Fig. 6d and Supplementary Fig. 8d).

### Administration of CXCL1/2 restores insulin resistance in dAKO

**Fig. 7:**
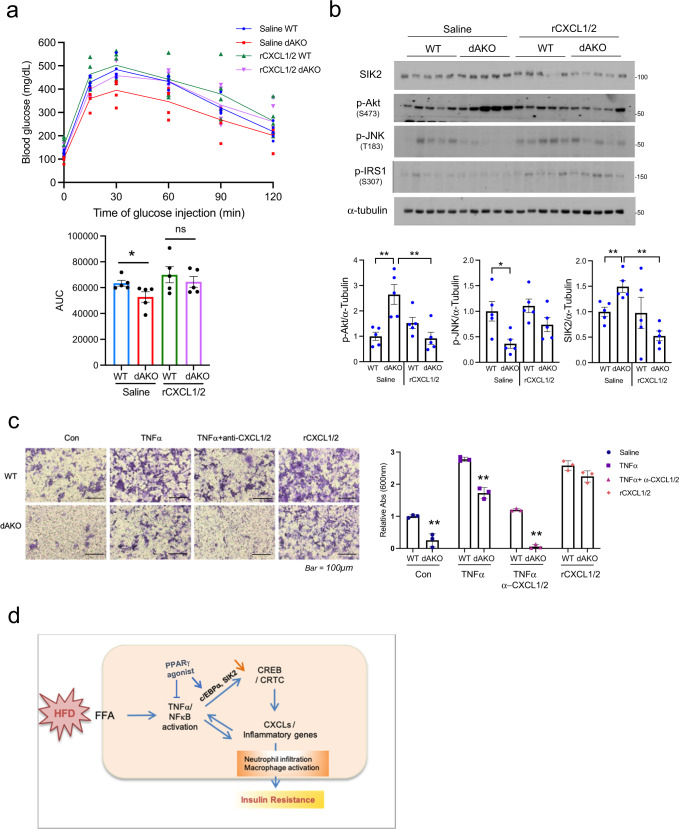
CXCL1/2 administration reverses salutary effects of adipocyte CRTC2/3 depletion on glucose homeostasis and pro-inflammatory gene expression. **a** Effect of recombinant CXCL1/2 (rCXCL1/2) on CRTC dAKO mediated glucose tolerance following 12 weeks of HFD (1 g/kg of glucose). 1 µg of rCXCL1 and 2 were administered by IP injection 1 week prior to the IPGTT assay. Bottom, AUC of glucose tolerance test (*P < 0.05, one-way ANOVA, *n* = 5 per group). **b** Immunoblots of eWAT showing the effect of rCXCL1/2 on insulin and stress signaling as well as SIK2 protein amounts in eWAT from HFD-fed (12 weeks) WT and dAKO mice (*n* = 5 per group). Bottom, densitometric analysis of p-Akt, p-JNK and SIK2 expression (**P < 0.01, *P < 0.05, one-way ANOVA; *n* = 5 per group). **c** Effect of TNFα, α-CXCL1/2, and rCXCL1/2 on LPS-primed Raw264.7 migration. Adipocytes from WT and dAKO mice were treated overnight with 10 ng/ml TNFα, 100 ng/ml α-CXCL1/2, and 100 ng/ml rCXCL1/2. Following 16 h exposure to the adipocyte conditioned medium, Raw 264.7 macrophage migration was evaluated by staining inserts with crystal violet. Dried inserts were photographed and dyes were extracted with alcohol/acetic acid and quantified by OD measurement at 600 nm. Bottom, relative absorbance shown (**P < 0.01, *t*-test; *n* = 3 per group). Data in **a**–**c** represent the mean ± SEM. **d** Diagram showing the effect of HFD feeding on CRTC2/3 activation and induction of cytokine genes in cooperation with NF-κB.

We wondered whether CXCL1/2 expression is sufficient to promote glucose intolerance and insulin resistance. We tested this notion by evaluating the effects of recombinant CXCL1/2 proteins (rCXCL1/2) on adipose tissue. Intra-peritoneal injection of rCXCL1/2 reversed the salutary effects of CRTC2/3 depletion in WAT (Fig. 7a–c and Supplementary Fig. 9). Indeed, rCXCL1/2 administration blocked dAKO-dependent increases in insulin signaling (p-Akt) and stimulated increases in stress signaling (p-JNK) that lead to inhibitory phosphorylation of IRS1 (at Ser307) (Fig. 7d). Taken together, these results indicate that CXCL1/2 are important downstream components of the CREB/CRTC-mediated response to HFD feeding in WAT.

To address the potential role of CXCL1/2 in promoting the migration of innate immune cells, we performed in vitro chemotaxis assays using the macrophage/monocyte cell line Raw 264.7. Exposure to conditioned medium from unstimulated WT adipocytes promoted macrophage migration (Fig. 7c); these effects were further potentiated by exposure to conditioned medium from WT adipocytes treated with TNFα. dAKO adipocyte conditioned medium was far less effective in promoting macrophage migration, under basal conditions and following TNFα treatment. TNFα-inducible macrophage chemotaxis was partially blocked by the addition of CXCL1/2 neutralizing antiserum. Conversely, the addition of rCXCL1/2 increased macrophage migration even in conditioned medium from dAKO cells. Taken together, these results demonstrate that CXCL1/2 acts downstream of CREB/CRTC and NFκB pathways to promote insulin resistance in obesity.

## Discussion

Obesity is a major risk factor for the development of insulin resistance and type II diabetes. High-fat diet feeding stimulates the release of cytokines from adipocytes, which in turn recruit innate immune cells that disrupt fat storage and block insulin signaling^4^. TNFα and other cytokines are thought to mediate the effects of HFD in promoting the expression of inflammatory genes and by inhibiting metabolic gene expression via the induction of NF-κB^21,45^. We found that the NF-κB pathway stimulates a subset of inflammatory genes cooperatively with CREB/CRTC. Although CREB and CRTCs are typically activated by cAMP, HFD feeding appears to induce this pathway in adipocytes by decreasing the expression of SIK2.

Indeed, SIK2 expression and function are inversely correlated with insulin resistance and obesity in humans^12,29^. While inhibition of SIK2 in macrophages appears to promote the release of anti-inflammatory cytokines^48^, adipocyte SIK2 has been found to regulate Glut4 expression and insulin signaling^28^. Correspondingly, SIK2^−/−^ mice have lower glucose tolerance and reduced insulin sensitivity, due to increases in macrophage infiltration^14^. The extent to which CRTCs contribute to this phenotype is unclear, however.

Although it disrupts adipocyte cAMP signaling, HFD feeding paradoxically activates the CREB/CRTC pathway, in part via cytokine-mediated decreases in SIK2 expression that lead to CRTC dephosphorylation, nuclear entry, and association with CREB over relevant promoters. Restoring SIK2 expression in adipocytes, by overexpression of C/EBPα or by administration of PPARγ agonist, rescued the inhibitory phosphorylation of the CRTCs. Future studies should reveal the extent to which PPARγ may also modulate CREB/CRTC activity in other tissues.

Following their induction in response to HFD feeding, adipocyte CRTC2/3 stimulates the expression of CXCL1 and CXCL2 as well as other pro-inflammatory genes (IL6, LIF) in cooperation with NFκB. In turn, CXCL1/2 recruit neutrophils and macrophages to adipose tissue, where they promote insulin resistance (Fig. 7d). Loss of CXCL1/2 expression, by immune-depletion or by knockout (of CXCL1), restored insulin sensitivity and glucose tolerance. Indeed, these chemokines have also been shown to function importantly in human obesity and insulin resistance^39,42,44^ as well as in cancer metastasis^49,50^.

Although they are indistinguishable from wild-type littermates under NCD conditions, dAKO mice weigh less and have lower fat mass than wild-type littermates in response to HFD feeding. In this regard, CREB has been reported to promote the expression of the adipogenic program by stimulating the expression of the c/EBPβ gene^51^. c/EBPβ mRNA levels tend to be decreased in dAKO WAT but adipogenic gene expression appears to be relatively unaffected, likely reflecting compensation by the closely related family member c/EBPδ^52^. c/EBPα and PPARγ protein levels are elevated in dAKO relative to wild-type WAT. And late adipogenic markers (e.g., FABP4, LPL, PEPCK) are either equally expressed or upregulated in dAKO WAT. Further studies should reveal how crosstalk between adipogenic and inflammatory pathways contributes to the development of insulin resistance.

In addition to their effects on triglyceride storage in WAT, pro-inflammatory cytokines have been shown to interfere with thermogenesis in beige and brown fat^53^. We imagine that the loss of pro-inflammatory (CXCL1, CXCL2) gene expression in dAKO mice could therefore enhance the expression of thermogenic genes, either in beige adipocytes or in brown fat. Indeed, oxygen consumption and energy expenditure are elevated in dAKO mice. Moreover, CXCL1 KO mice have reduced body weight and tend to have lower fat mass compared to wild-type littermates (Fig. 6a). Thermogenesis may be more active in dAKO mice, although the modulatory effects of CRTC3 alone in brown fat appear to be largely developmental^54^.

Knockout of CRTC2/3 not only reduces CXCL1/2 expression but also blunts the effects of NFκB activation on pro-inflammatory genes. These results suggest that CRTCs may prime certain promoters for NFκB recruitment through epigenetic mechanisms. CRTC2/NFκB co-bound peaks appear to be particularly enriched in AP1 binding sites, which are typically recognized by Jun/Fos but not CREB family members. Supporting this idea, CRTC1 has been reported to associate with AP1 and to mediate induction of target gene expression^47^. Future studies should reveal the extent to which CRTCs modulate adipose tissue function through other nuclear factors in addition to CREB.

## Materials and methods

### Animals

All procedures involving the use of animals were performed in accordance with the guidelines presented by Salk Institute’s Animal Care and Use Committee. C57BL/6J mice were purchased from The Jackson Laboratory (strain #000664). CRTC2/3 double-floxed (dflox) mice were produced by crossing Crtc2-floxed mice^55^ with Crtc3-floxed mice^54^. Adipose-specific CRTC2/3 knockout (dAKO) mice were generated by crossing CRTC2/3 dflox mice with *Adiponectin*-Cre mice (Jackson Laboratory; strain # 010803). CXCL1 knockout mice, C57BL/6NCrl-*Cxcl1*^*em1(IMPC)Mbp*^/Mmucd, RRID: MMRRC_046310-UCD, were obtained from the Mutant Mouse Resource and Research Center (MMRRC) at the University of California at Davis, an NIH-funded strain repository, and were donated to the MMRRC by Kent Lloyd, D.V.M., University of California, Davis.

For High Fat Diet (HFD) feeding, 4–6-week-old mice were fed 60% HFD (Research Diet # D12492) for indicated periods with regular day/night cycles. For glucose tolerance testing (GTT), mice fed with a 60% high-fat diet were fasted overnight and injected with 1 g/kg of glucose/body weight. Blood glucose levels were monitored at indicated time points for 2 h. For insulin tolerance testing (ITT), mice were fasted for 4 h and injected with 1 U/kg of insulin/body weight. Blood glucose levels were determined at indicated time points. Body fat composition was analyzed by EcoMRI-100H.

### Histology

Mouse tissues were fixed in zinc-buffered formalin (Anatech) and paraffin-embedded. Sections (5–10 mm) were used for hematoxylin and eosin (H&E) staining or immunohistochemistry. For immunohistochemical staining of epididymal WAT (eWAT), sections were rehydrated and antigen-retrieved in sodium citrate. The sections were incubated with F4/80 (Abcam) or Ly6G (Biolegend) antiserum and visualized by the avidin–biotin-complex method using the chromogen diaminobenzidine (Vector Labs). Stained slides were imaged by light-field microscopy (Nikon).

### Indirect calorimetry

Mice fed with a 60% high-fat diet for 3 months were individually housed for at least 3 days before experiments. Food intake, locomotor activity, oxygen consumption, and energy expenditure were simultaneously measured for individually housed mice with a LabMaster system (TSE Systems). Data were collected for 2–3 days and analyzed.

### Primary adipocyte and 3T3-L1 cells culture

Primary adipocytes were generated and maintained as previously reported^54^. Interscapular BAT and eWAT were collected, minced, and digested with isolation buffer for a proper time at 37 °C on a shaker. The isolation buffer contains 1.5 mg/ml Collagenase I (for eWAT) or 1 mg/ml Collagenase IV (for BAT). After digestion and filtration, stromal vascular fraction (SVF) cells were cultured in a growth medium for 3 days, and then fresh media was changed every 2 days. Upon confluence, cells were exposed to an induction medium for 2 days and then a differentiation medium. After differentiation, cells were considered primary adipocytes. The induction medium contains DMEM (Mediatech), 10% FBS (Gemini), 1 µg/ml insulin (HumulinR; Lilly), 0.3 μM dexamethasone (Sigma) and 0.63 mM 3-isobutyl-1-methylxanthine (IBMX) (Sigma), 1 µM Rosiglitazone (Adipogen), 10 nM T3 (Sigma). The differentiation medium contains DMEM, 10% FBS, 1 µg/ml insulin, and 10 nM T3. For 3T3-L1 differentiation, we employed the same procedure as for primary adipocytes. Subcellular fractionation for TNFα treated 3T3-L1 adipocytes was performed using a NE-PER Nuc-Cyt extraction kit according to the manufacturer’s protocol (Thermo Scientific).

### Immunofluorescence staining

Immunofluorescence staining was performed as previously described^54^. eWAT from HFD (6 weeks) and age-matched NCD mice were fixed with ice-chilled 4% PFA for 3 h, stored overnight in 25% sucrose solution, and then embedded in OCT compound. After cutting at 15–30 micrometer using cryostat, slides were blocked for 1 h in blocking buffer containing PBS, 3% normal goat serum, 2% bovine serum albumin, and 0.2%Triton X-100. The sections were incubated with anti-CRTC3 antibody (Cell signaling technology) or anti-CRTC2 antibody (generated in our laboratory) or anti-F4/80 (Abcam) diluted in blocking buffer overnight at 4 °C. Slides were washed three times with PBS containing 0.2% Triton X-100, and then incubated with secondary antibodies and DAPI (Vector Laboratories) for 1 h at room temperature, mounted and images were taken under Zeiss750 confocal microscope.

### Luciferase assay

Plasmid carrying a ~600 base pair fragment of CXCL1-proximal promoter in pGL3 luciferase vector was transfected into HIB1b brown pre-adipocytes, along with control, CRTC2, CRTC3, or NFκB p65 and RSV-β gal plasmids. Mouse SIK2 promoter (~910 bp) was cloned into pGL4 luciferase vector and mouse C/EBPα promoter (~1350 bp) was cloned in pXP2 luciferase vector; promoter assay was performed in HIB1B cells. Plasmid carrying a −345 base pairs of human-IL8 proximal promoter in pXP2 luciferase vector were transfected to 293T cells, along with control, CRTC2, CRTC3 or NFκB p65 and RSV-β gal plasmids. Samples were collected 48 h post-transfection and luciferase activity was measured by GloMax luminometer (Promega). Luciferase activity was normalized to β-gal activity. Forskolin (FSK), murine TNFα, human TNFα were purchased from Sigma, Novus Biologicals and MyBiosource, respectively.

### Flow cytometry

The stromal vascular fraction of epididymal adipose tissues were isolated as described above. Cells were stained for 1 h and washed three times with HBSS containing 1% FBS. The macrophages (F4/80^+^CD11b^+^) and neutrophils (F4/80-Ly6g^+^CD11b^+^) were analyzed by FACS LSRII (BD Biosciences). The antibodies were purchased from Serotec (F4/80) and BD Biosciences (CD11b and Ly6g). For the collection of endothelial/immune cells, stained cells with CD31, CD45, Ter119 were collected by FACS Aria (BD Biosciences). The antibodies were obtained from eBioscience. The gating strategy is described in Supplementary Data.

### Lentivirus production

Lenti-mouse c/EBPα was prepared by cloning c/EBPα cDNA into the pHRST vector. pHRST-control, pHRST-c/EBPα along with packaging plasmids were transfected in HEK293 cells. After 3 days of incubation, amplified virus particles were collected and concentrated.

### RNA analysis

Total RNA was extracted from cells or tissue with TRIzol-based isolation Kits (Zymo Research) and 1 μg of RNA was converted to cDNA with Transcriptor first-strand cDNA synthesis kit according to the manufacturer’s protocol (Roche). Quantitative PCR (qPCR) was performed with SYBR green master mix (Roche) by Light cycler 480 II (Roche) and the relative mRNA expression was calculated by 2^−ΔΔct^ method. *L32* was used as a housekeeping gene for mRNA expression analysis. RNA-seq was performed as previously described^54^. RNA-Seq libraries were prepared following the manufacturer’s protocols using the NEB Next-poly(A) magnetic isolation kit to isolate mRNA followed by the NEBNext-Ultra2 kits from New England Biolabs. Libraries were quantitated by Qubit (Invitrogen), and run on a MiSeq instrument with paired-end 75 bp reads using v3 chemistry (Illumina). Data were analyzed by tophat2 and cuffdiff against the mouse mm10 genome build. GEO accession number for RNA-seq studies in Fig. 1a, b and Supplementary Fig. 7d is GSE160684. RNA-seq data for Supplementary Fig. 2a was taken from GEO accession number GSE109443^54^.

### Primer sequences for qRT-PCR

L32: F-5′ TCT GGTGAAGCCCAAGATCG-3′, R-5′ CCTCTGGGTTTCCGCCAG TT-3′

CRTC2: F-5′ GTGGTTCTCTGCCCAATGTT-3′, R-5′ CAGACTCTGGGGGAGGAGAT-3′

CRTC3: F-5′ CAAGCCGATAATGTTCGTGGAAC-3′, R-5′ CTGTGAAGAATAACTGGCTGCG-3′

CRTC1: F-5′ TGCCCAACGTGAACCAGATT-3′, R-5′ GTCGCCCATGCTTGTCTACT-3′

CXCL1: F-5′ TGCACCCAAACCGAAGTCAT-3′, R-5′ ACTTGGGGACACCTTTTAGCA-3′

CXCL2: F-5′ TCA TAG CCA CTC TCA AGG GC-3′, R-5′ TCT TCC GTT GAG GGA CAG CA-3′

CXCL5: F-5′ CAGTGCCCTACGGTGGAAGT-3′, R-5′ GCGAGTGCATTCCGCTTAG-3′

SIK2: F-5′ TGTGGGGCTGCCAGTGACCT-3′, R-5′ AGGGGGCACAGGGTCAAGCA-3′

SIK3: F-5′GCAGGGTCCCTAGCCATTTT-3′, R-5′ CCACGTAAACAGGTCAGGCT-3′

c/EBPα: F-5′ TGGACAAGAACAGCAACGAG-3′, R-5′ TCACTGGTCAACTCCAGCAC-3′

ADRB3: F-5′ ACAGGAATGCCACTCCAATC-3′, R-5′ GGGGAAGGTAGAAGGAGACG-3′

Glut4: F-5′ GAT GGG GAA CCC CCT CGG CA-3′, R-5′ GGT CCC CCA GGA CCT TGC CT-3′

AdipoQ: F-5′ GGGCTCAGGATGCTACTGTT-3′, R-5′ ACCTGCACAAGTTCCCTTGG-3′

CD11b: F-5′ CCACACTAGCATCAAGGGCA-3′, R-5′ AAGAGCTTCACACTGCCACC-3′

F4/80: F-5′ GTGCCATCATTGCGGGATTC-3′, R-5′ GGAAGCCCATAGCCAAAGG-3′

IL6: F-5′ GGG ACT GAT GCT GGT GAC AA-3′, R-5′ TCC ACG ATT TCC CAG AGA ACA-3′

MCP1: F-5′ GTC CCT GTC ATG CTT CTG GG-3′, R-5′ GAG TAG CAG CAG GTG AGT GG-3′

c/EBPβ: F-5′ ACTTCAGCCCCTACCTGGAG-3′, R-5′ AGAGGTCGGAGAGGAAGTCG-3′

ATF3: F-5′ ACCGCCATTGTCCCCTGCCT-3′, R-5′ GGGGCCGCCTCAGACTTGGT-3′

JunB: F-5′ TCACGACGACTCTTACGCAG-3′, R-5′ GATAGGGATCCGCCAGGTTG-3′

PTGER3: F-5′ ATCCTCGTGTACCTGTCACAGCGA-3′, R-5′ TCAACCGACATCTGATTGAAGAT-3′

PTGER4: F-5′ TGT TCA TCT TCG GGG TGG TG-3′, R-5′ GCC ACT GGC CCT TCA TGT AT-3′

NR4A1: F-5′ GGA CAA GAG GCG GCG GAA CC-3′, R-5′ GGC CCG GAG TCC AAG TGT GC-3′

N-Elastase: F-5′ CGGCCTAAATTTCCGGTCAG-3′, R-5′ ACGTTGGCGTTAATGGTAGC-3′

TNFα: F-5′ AGG CAC TCC CCC AAA AGA TG-3′, R-5′ TGA GGG TCT GGG CCA TAG AA-3′

IL1β: F-5′ AAATGCCTC GTGCTGTCTGAC C-3′, R-5′ CTGCTTGAGAGG TGCTGATGTACC-3′

RelA: F-5′ GCGGAGCTTGTAGTCGGG-3′, R-5′ AGGGGAAACAGATCTGAAAGGG-3′

NFκB2: F-5′ CTGGTGGACACATACAGGAAGAC-3′, R-5′ ATAGGCACTGTCTTCTTTCACCTC-3′

Chil3: F-5′ ACTGGAAGGACCATGGAGCA-3′, R-5′ TAGGGGCACCAATTCCAGTC-3′

CD163: F-5′ TGCTGTCACTAACGCTCCTG-3′, R-5′ TCATTCATGCTCCAGCCGTT-3′

### Western blotting

Proteins were resolved on SDS-PAGE (Bio-Rad), transferred onto nitrocellulose membranes (Amersham), and probed with specific antibodies from Cell Signaling Technologies (CRTC3, pPKA substrate, c/EBPα, SIK2, HDAC4, Glut4, p-HSL S563, p-Akt S473, p-p38 T180/Y182, p-p65 S536, Ac-p65 K310, p-JNK T183/Y185, p-AMPK T172) Santa Cruz Biotechnology (c/EBPα, ATF3, JunB, HSP90), Millipore (SIK2, α-tubulin), LS Bio (ADRB3), Boster Bio (CD11b), Invitrogen (p-SIK2 Thr175, p-IRS1 S307), ABclonal (PTGER3) and homemade (CRTC2, CRTC3, p-CRTC2 S171, p-CRTC2/3 S275/273). For resolving CRTC3 phosphorylation, SDS-PAGE was performed using phos-tag according to the manufacture’s protocol (NARD Institute). Western blot densitometry was performed using Image J software.

### Chromatin-immunoprecipitation (ChIP)-sequencing

Chromatin-immunoprecipitation (ChIP) was performed as described previously^56^. ChIP-seq experiments for CRTC2 (homemade), NFκB p65 (Cell Signaling Technologies), and Histone H3AcK27 (H3KAcK27, Abcam) were conducted using 4-day differentiated 3T3-L1 adipocytes following 1 h of FSK, TNFα, and combined treatment. GEO accession number for ChIP-seq studies in Fig. 3e and Supplementary Fig. 4a–c is GSE160597.

### Neutralization and administration of CXCL1 and 2

For neutralization of CXCL1 and 2, 10 µg/mouse of anti-CXCL1 (KC), CXCL2 (MIP2), and control IgG antibody were introduced by retro-orbital injection. All antibodies were purchased from R&D systems. For recombinant protein administration, mice were injected 1 µg/mouse of recombinant murine CXCL1 and CXCL2 (Peprotech) or Saline intraperitoneally. IPGTT assay was conducted 1 week after each injection.

### LPS injection study

Low dose (500 ng/kg; Sigma-Aldrich) of LPS or an equal volume of saline was administered by intra-peritoneal injection. After 3 h of incubation, tissues were collected and snap-frozen in liquid nitrogen.

### Chemotaxis assay

Chemotaxis assay was performed with a slight modification of invasion assay^57,58^. Transwell inserts containing polycarbonate membrane (5 µm pore size; Corning costar) were preincubated in regular medium (DMEM supplemented with 10% FBS and 1% penicillin/streptomycin) for 30 min. Seeded LPS-primed (5 ng/ml, overnight) Raw264.7 murine monocyte cells (1 × 10^5^ cells in 500 µl serum-free medium) in the transwell insert and filled outside of insert with conditioned medium from WT and dAKO adipocytes following overnight treatment with saline, 10 ng/ml TNFα, TNFα + α-CXCL1/2 antisera (0.5 µg/ml) and rCXCL1/2 (0.1 µg/ml). After overnight incubation, aspirated medium and removed non-migrated cells in the upper membrane of an insert with a cotton swab. Then, washed insert and fixed in 4% formaldehyde for 10 min and stained with 0.1% crystal violet for 30 min. Following three washes with PBS and dry membrane, filters were photographed and dye was eluted in 50% ethanol plus 1% acetic acid and quantitated by measuring absorbance at 600 nm (GloMax multi detection system; Promega Inc).

### ELISA

Levels of CXCL1 and CXCL2 in serum and conditioned media were measured by the ELISA kit according to the manufacturer’s protocol (R&D Systems). Circulating and hepatic triglyceride and free fatty acids were analyzed by Elisa kit according to the manufacture’s protocol (BioVision).

### Statistics and reproducibility

The data were analyzed using Microsoft Excel and Graphpad PRISM8. The data are presented with mean ± SEM or mean ± SD. *P*-values were calculated using unpaired two-tailed student’s *t*-test and one-way ANOVA with post-hoc Tukey HSD test. Statistical significance was indicated as **P < 0.01 and *P < 0.05. A *P*-value of <0.05 was considered statistically significant.

### Reporting summary

Further information on research design is available in the Nature Research Reporting Summary linked to this article.

## Supplementary information

Supplementary Information

Description of Additional Supplementary Files

Supplementary Data

Reporting Summary

## Data Availability

The raw data for the main figure are available in Supplementary Data (graphs; Figs. 1–7) and Supplementary Fig. 10 (Uncropped Immunoblots; Figs. 1, 2, 5, 6, 7). GEO accession number for RNA-seq studies in Fig. 1a, b and Supplementary Fig. 7d is GSE160684. RNA-seq data for Supplementary Fig. 2a was taken from GEO accession number GSE109443^54^. GEO accession number for ChIP-seq studies in Fig. 3e and Supplementary Fig. 4a–c is GSE160597. Additional details can be obtained from the corresponding author on reasonable request.
