# Supplementary material for: Activation of the adipocyte CREB/CRTC pathway in obesity
Source: Commun Biol. 2021 Oct 22;4:1214. doi: 10.1038/s42003-021-02735-5 (PMC8536733; doi:10.1038/s42003-021-02735-5)
Supplement: Supplementary file 1 — Supplementary Information [file 42003_2021_2735_MOESM1_ESM.pdf]

Supplementary information

Activation of the Adipocyte CREB/CRTC Pathway in Obesity

Young-Sil Yoon[1], Weiyi Liu[1], Sam Van de Velde[1], Shigenobu Matsumura[2], Ezra Wiater[1], Ling Huang[3], Marc Montminy[1*]

[1]Peptide Biology Laboratories, The Salk Institute for Biological Studies, La Jolla, CA 92037, USA

[2]Department of Clinical Nutrition, Osaka Prefecture University, Habikino, Habikino-city, Osaka, Japan

[3]The Razavi Newman Integrative Genomics and Bioinformatics CoreThe Salk Institute for Biological Studies, La Jolla, CA 92037, USA

Supplementary Figure 1-10

Supplementary Table 1

Supplementary Data (Supplemental item 1-2: FACS Gating strategy)

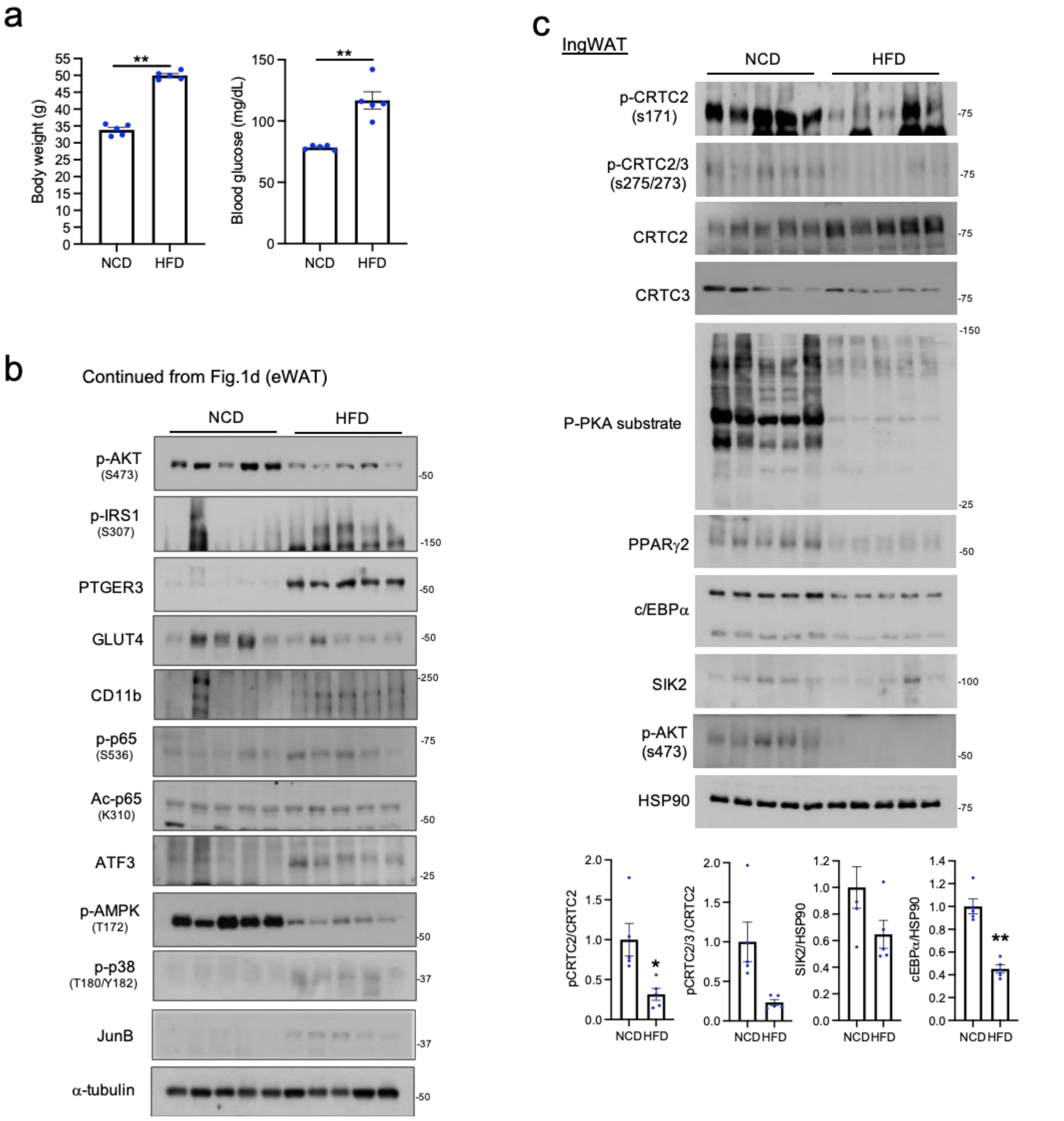

Supplementary Fig 1. **PKA activity in WAT from diet induced obese mice.**
a. Body weight (left) and blood glucose concentrations of overnight fasted C57BL/6J mice maintained on NCD or 15weeks HFD (**P<0.01, t-test; n=5 per group). **b.** Western blotting analysis of eWAT from age-matched NCD and HFD fed (15 weeks) mice. Data are continued from Fig 1d. **c.** Western blotting analysis of inguinal WAT (iWAT) from age-matched NCD and HFD fed (15 weeks) mice. Samples were prepared from mice in Fig 1d. Each lane represents a sample of one animal. Bottom, densitometry analysis showing decreased CRTC phosphorylation, c/EBPα, SIK2 expression (**P<0.01, *P<0.05, t-test; n=5 per group).

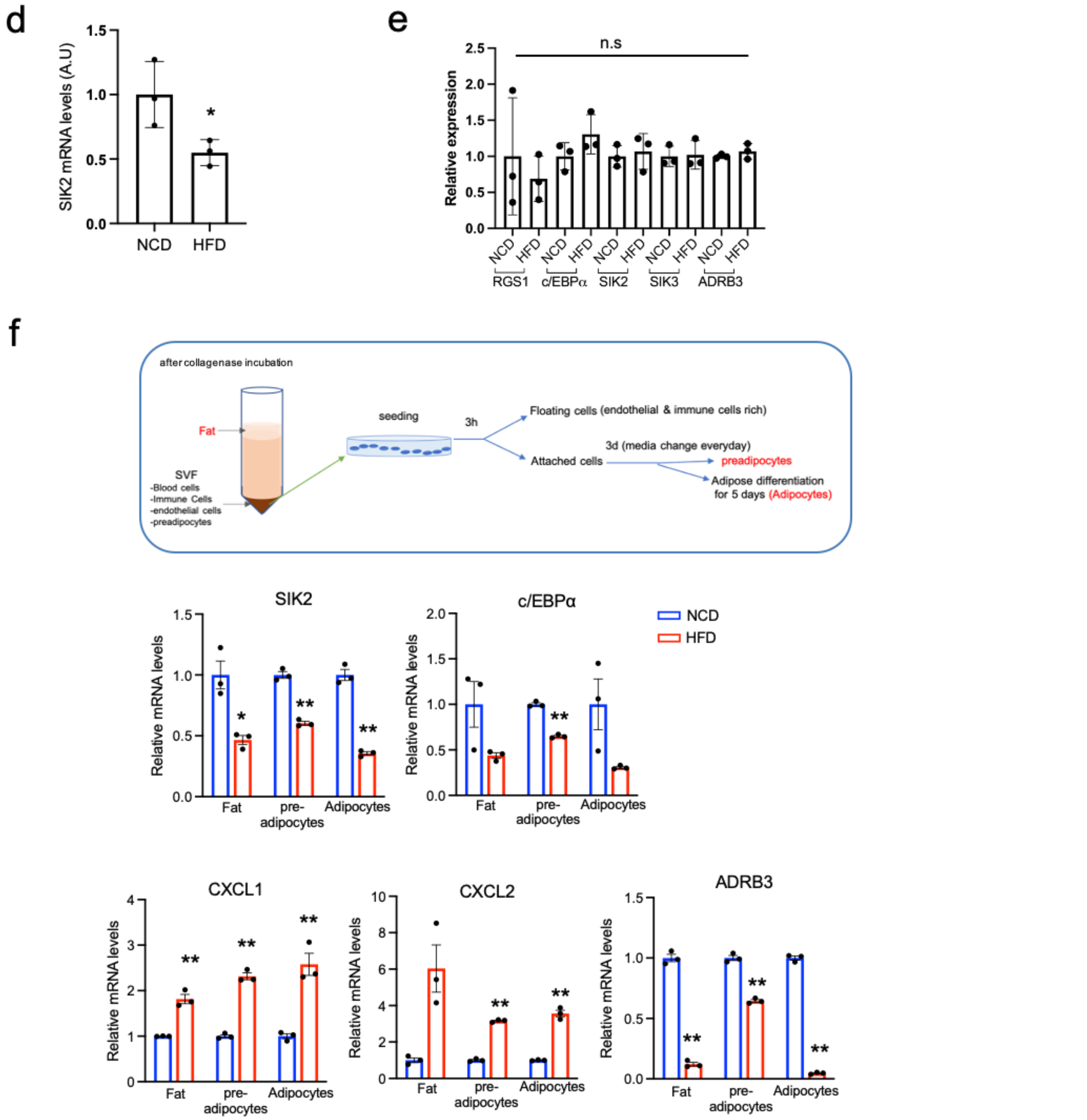

(continued)

d. q-PCR analysis of SIK2 expression in eWAT from age matched NCD and HFD fed (6weeks) mice in Fig.1e (*P<0.05, t-test; n=3 per group). **e.** qPCR analysis showing gene expression in endothelial/ hematopoietic ($CD31^+$; $CD45^+$; $Ter119^+$) cells isolated from eWAT of NCD and 15 weeks HFD fed mice (t-test; n=3 per group). **f.** Top, Schematic diagram for isolating adipose cells. Bottom, qPCR analysis showing effect of HFD (15weeks) on gene expression in each cell fractions (**P<0.01, *P<0.05, t-test; n=3 per group). Data in **a, c-d** represent the mean ± SEM and data in **e-f** represent the mean ± SD.

a

Brown pre-adipocytes

	gene ID	con	Fsk	log2(fold_change)	q_value
1	Nr4a2	1.8	64.1	5.1	0.0137175
2	Crem	7.0	187.0	4.7	0.0137175
3	Gem	3.7	78.5	4.4	0.0137175
4	Dgat1	8.4	169.0	4.3	0.0137175
5	Star	4.6	88.0	4.2	0.0137175
6	Dio2	1.4	22.9	4.0	0.0137175
7	Vdr	17.4	268.9	3.9	0.0137175
8	Nfil3	6.2	78.5	3.7	0.0137175
9	Tnfaip6	6.3	78.7	3.6	0.0137175
10	Uap1	55.1	615.9	3.5	0.0137175
11	Kcne4	20.9	232.5	3.5	0.0137175
12	Slc25a25	3.3	36.1	3.5	0.0137175
13	Tuba4a	11.3	119.2	3.4	0.0137175
14	Pde4d	4.3	45.0	3.4	0.0137175
15	Procr	13.6	136.5	3.3	0.0137175
16	Gfpt2	28.6	286.4	3.3	0.0137175
17	Cxcl1	13.6	121.1	3.2	0.0137175
18	Ier3	37.5	319.9	3.1	0.0137175
19	Nmrk1	6.0	49.6	3.0	0.0415619
20	Adamts4	4.9	39.4	3.0	0.0137175

20 genes out of 23406 genes

b

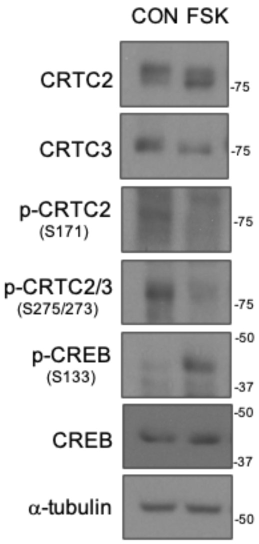

Supplementary Fig 2. **CRTC dependent gene expression in primary pre-adipocytes**.
a. RNA-seq analysis of primary brown pre-adipocytes showing top 20 genes that are up-regulated more than 8-fold (log FC>3) following exposure to FSK (10μM FSK, 1hr). **b.** Immunoblots showing CRTC2 and CRTC3 activation in primary brown pre-adipocytes following FSK treatment.

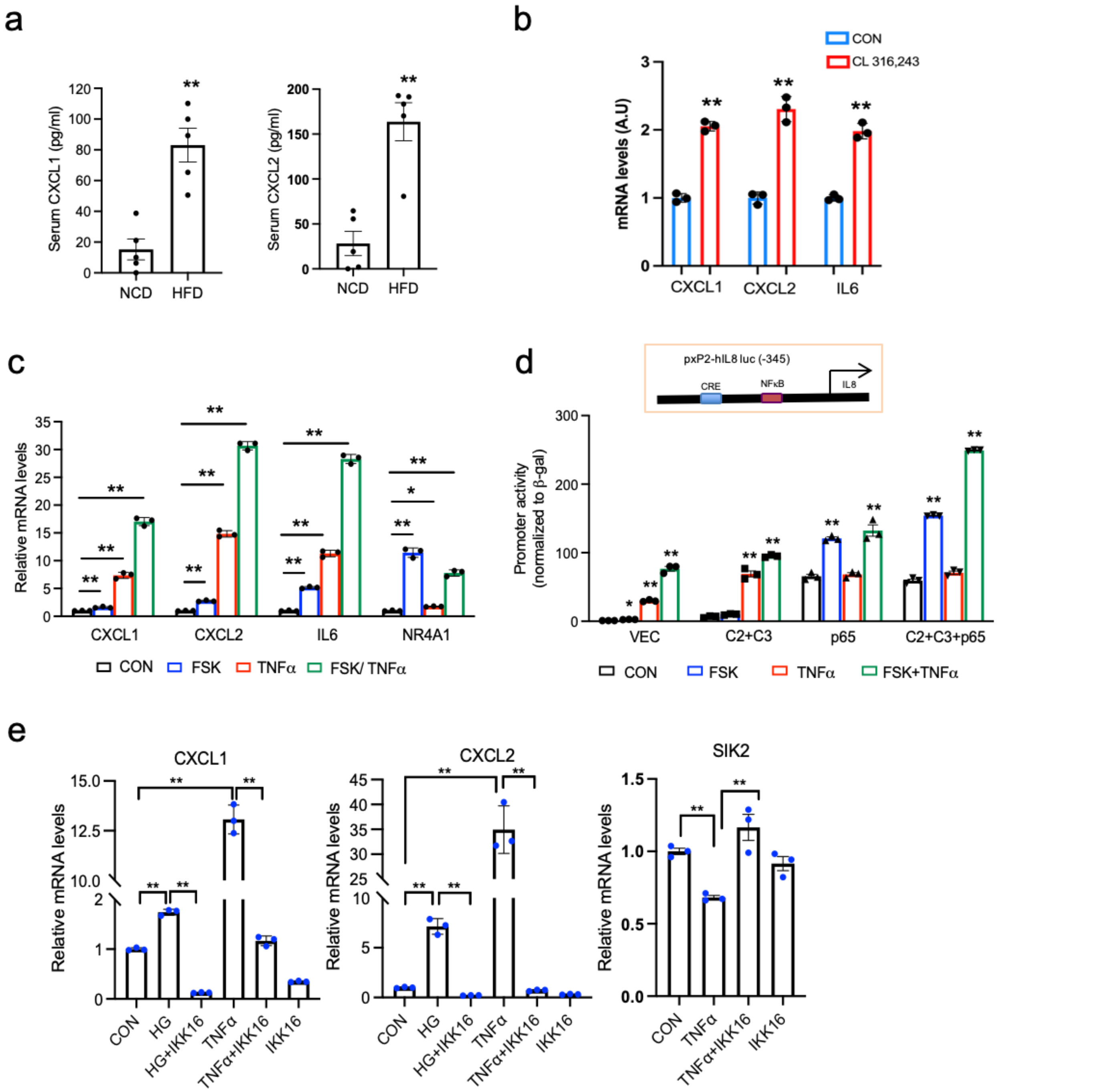

Supplementary Fig 3. **Regulation of cytokine genes by cAMP and TNFα.**
a. Circulating concentrations of CXCL1 and CXCL2 maintained on a NCD or HFD for 15 weeks (**P<0.01, t-test; n=5 per group). **b.** Effects of β3 adrenergic agonist CL316,243 on inflammatory gene expression in primary white adipocytes. All cells were differentiated for 7days before exposure to CL316,243 (10 μM, 1hr) (**P<0.01, t-test; n=3 per group). **c.** Effect of FSK and TNFα (2hrs) alone and together on cytokine gene expression in differentiated 3T3L1 cells. Effect of FSK and TNFα on a CREB target gene (NR4A1) that lacks an NF-κB binding site (**P<0.01, *P<0.05, one-way ANOVA; n=3 per group). **d.** Cooperative effects of CRTC2/3 and p65 on human CXCL8 (IL8) expression. Relative effect of CRTC2 (C2), CRTC3 (C3) and p65 on human IL8 promoter activity in 293T cells shown. Luciferase activity normalized to RSV-β-gal activity (**P<0.01, *P<0.05, one-way ANOVA; n=3 per group). **e.** Effect of NFκB signaling blockage on TNFα (10ng/ml, 2hrs) or HG9-91-01 (10μM, 2hrs) induced CXCLs expression and TNFα (10ng/ml, 6hrs) induced SIK2 expression in white adipocytes. IKK inhibitor IKK16(10μM) was treated 30min prior to treatment with TNFα or HG9-91-01 (**P<0.01, one-way ANOVA; n=3 per group). Data in **a.** represent the mean ± SEM and **b-e** represent the mean ± SD.

a

KEGG_pathway	Term	Count	%	P_Value	FDR
mmu05222	Small cell lung cancer	11	3.343465	8.31E-07	1.35E-04
mmu04668	TNF signaling pathway	11	3.343465	9.14E-06	7.40E-04
mmu04380	Osteoclast differentiation	11	3.343465	3.28E-05	0.00177086
mmu04512	ECM-receptor interaction	9	2.7355623	7.96E-05	0.00322315
mmu04510	Focal adhesion	13	3.9513678	1.18E-04	0.00349352
mmu04060	Cytokine-cytokine receptor interaction	14	4.2553191	1.32E-04	0.00349352
mmu04151	PI3K-Akt signaling pathway	17	5.1671733	1.51E-04	0.00349352
mmu04621	NOD-like receptor signaling pathway	7	2.1276596	2.88E-04	0.00583738
mmu04210	Apoptosis	7	2.1276596	3.82E-04	0.00688225
mmu05200	Pathways in cancer	17	5.1671733	5.71E-04	0.00924298
mmu04064	NF-kappa B signaling pathway	8	2.4316109	9.24E-04	0.01360811
mmu05145	Toxoplasmosis	8	2.4316109	0.00147249	0.01987865
mmu05323	Rheumatoid arthritis	7	2.1276596	0.00200541	0.02499046

b

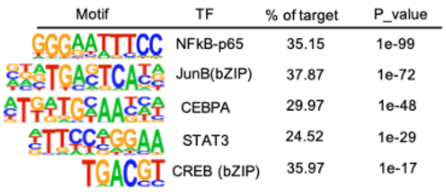

c

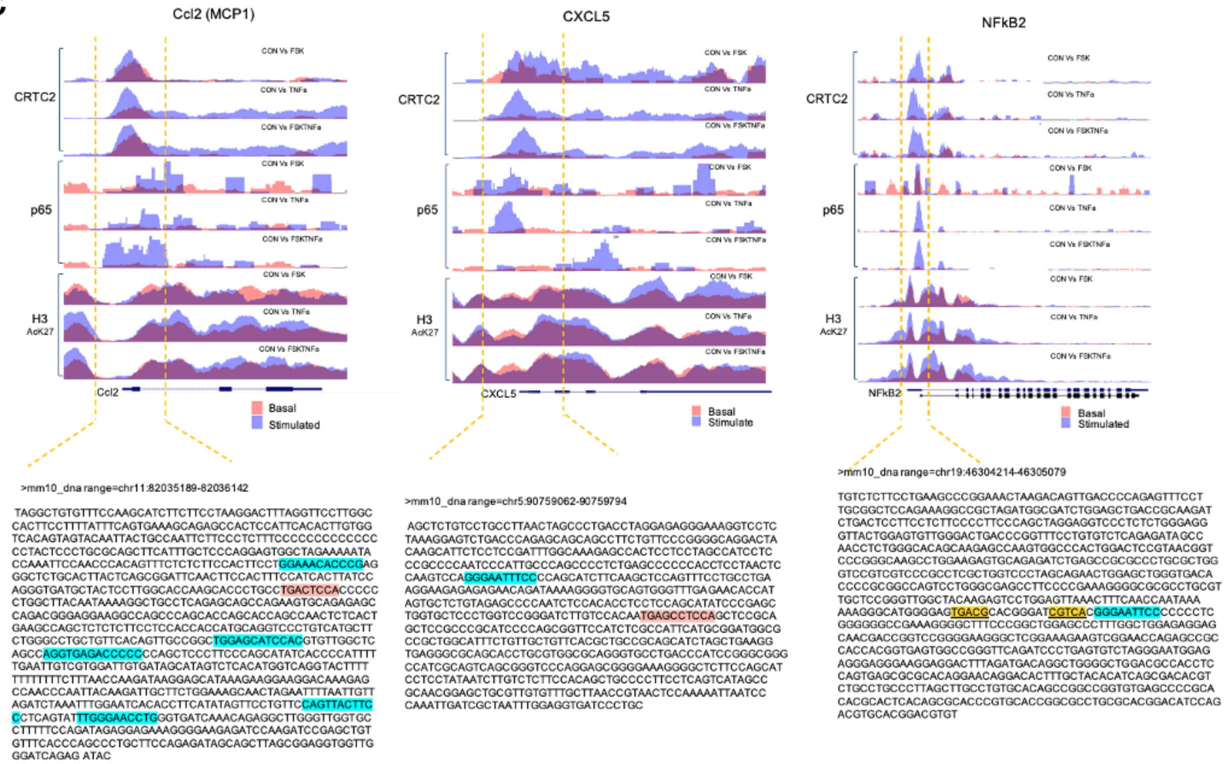

d

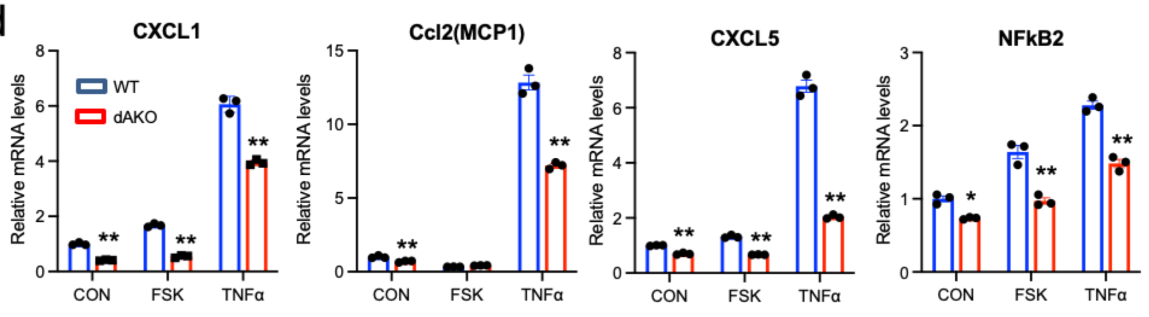

Supplementary Fig 4. **Regulation of CRTC2 and NF-κB co-occupancy.** **a**. Gene ontology analysis for CRTC2-p65 co-bound loci following 1hr exposure of 3T3-L1 adipocytes to TNFα and FSK. **b.** HOMER motif analysis of CRTC2- p65 co-bound loci showing top 5 binding motifs after 1hr exposure to TNFα and FSK (10μM). **c.** Genome browser plots showing ChIP-seq analysis of CRTC2, p65, and H3AcK27 occupancy over the mouse CCL2, CXCL5, and NFκB2 promoters in 4 day-differentiated 3T3-L1 adipocytes. Gene sequences corresponding to indicated peak area are shown. Blue, yellow, and red highlighting corresponds to putative NF-κB, CREB, and JUN/bZIP binding sites, respectively. **d**. mRNA amounts for NF-κB target genes in 5d differentiated white adipocytes from dAKO and WT mice following exposure to FSK (10μM) or TNFα (10ng/ml) for 1hr (**P<0.01, *P<0.05, t-test; n=3 per group). Data in **d** represent the mean ± SD.

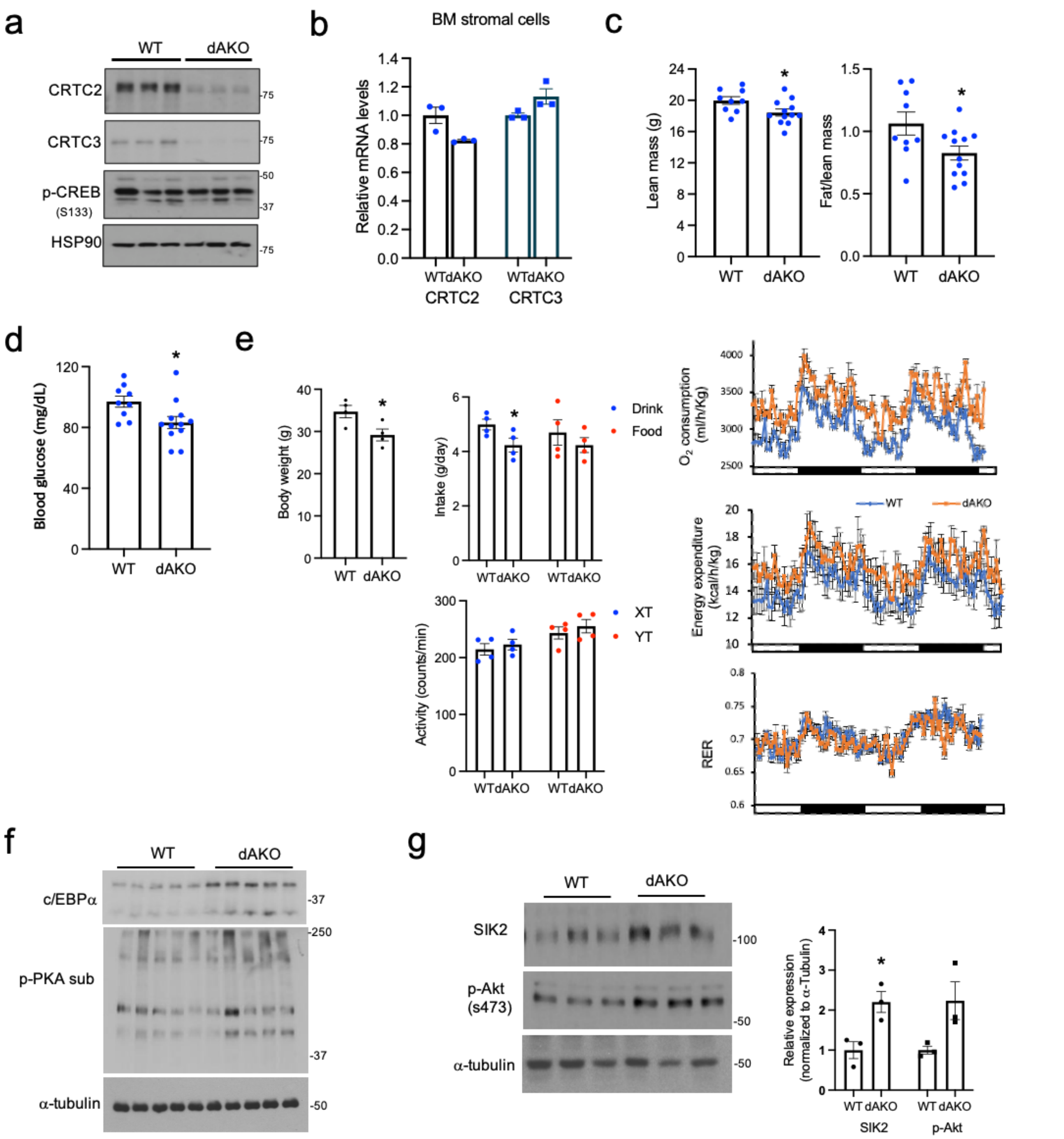

Supplementary Fig 5. Characterization of CRTC2/3 dAKO mice. a. Immunoblot analysis of CRTC2 and CRTC3 protein amounts in eWAT from dAKO and WT littermates after 12 weeks of HFD. **b.** mRNA amounts for CRTC2 and CRTC3 in bone marrow stromal cells from WT and dAKO mice. **c.** Body composition of dAKO and WT littermates (*P<0.05, t-test; n=9, 12 per group). Data are continued from Fig 4a. **d.** Blood glucose concentrations in dAKO and WT littermates after fasting for 18hrs (*P<0.05, t-test; n=9, 12 per group). Mice were fed an HFD for 12 weeks. **e.** Increased energy expenditure in dAKO mice. Indirect calorimetric analysis conducted for 48hrs following 72hrs adaptation in individual cages (*P<0.05, t-test; n=4 per group). Mice were fed an HFD for 9 weeks. **f**. Immunoblot analysis showing effect of CRTC2/3 depletion on PKA substrate phosphorylation and c/EBPα expression. Mice were fed an HFD for 12 weeks. Each lane represents one mouse sample. **g**. Immunoblot showing effect of CRTC2/3 depletion on p-Akt (Ser 473) and SIK2 protein amounts in primary adipocytes from dAKO and WT littermates. Right, densitometry analysis for immunoblots shown (*P<0.05, t-test; n=3 per group). Data in **b.** represents the mean ± SD and **c-e, g** represent the mean ± SEM.

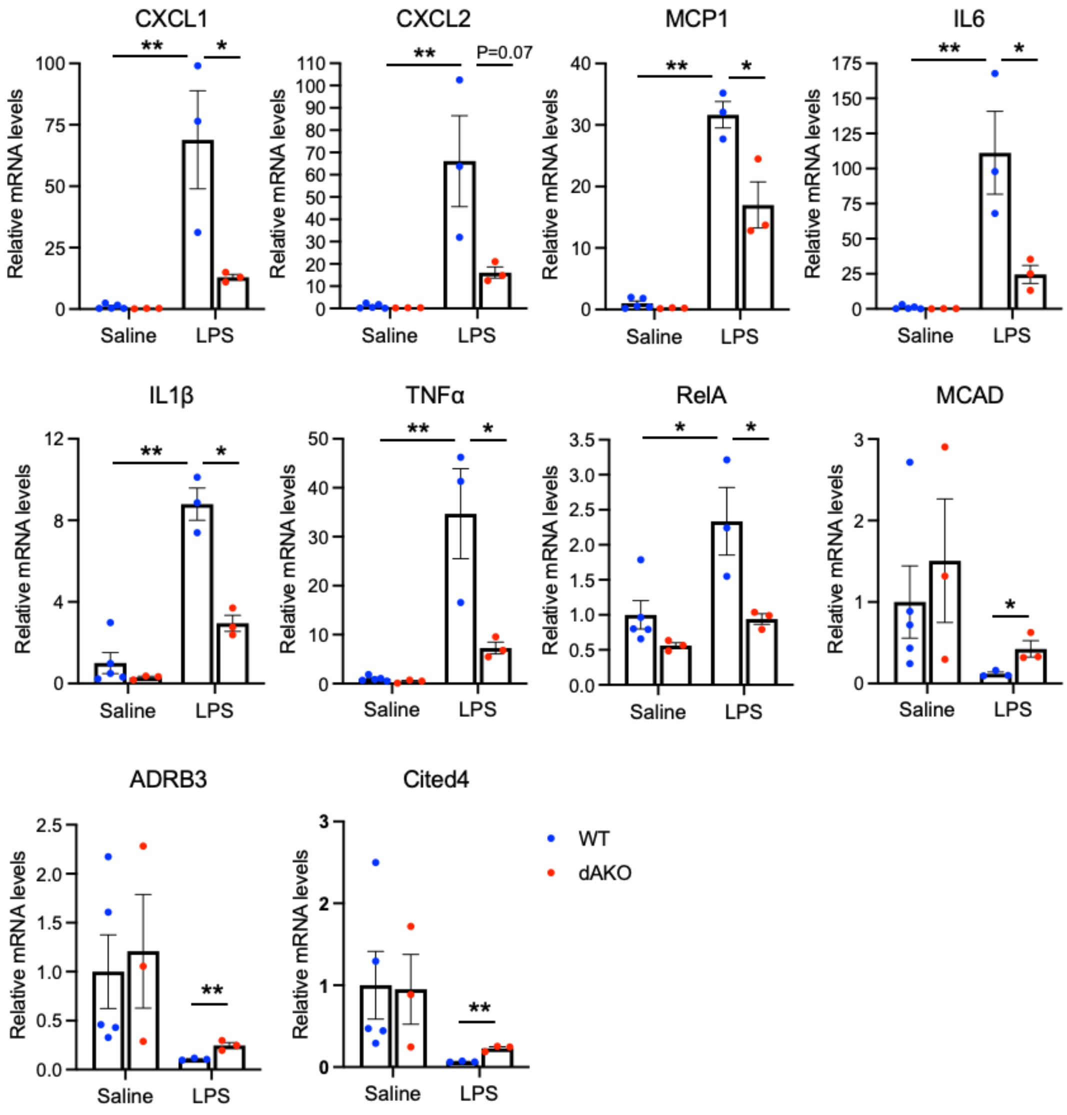

Supplementary Fig 6. **Downregulation of LPS-inducible inflammatory gene expression in dAKO WAT.**

mRNA amounts for immune-related genes in eWAT from dAKO and WT littermates following administration of low dose LPS (500ng/kg) for 3hrs in 10 weeks old mice maintained on an NCD (**P<0.01, *P<0.05, t-test; n=3~5). Data represent the mean ± SEM.

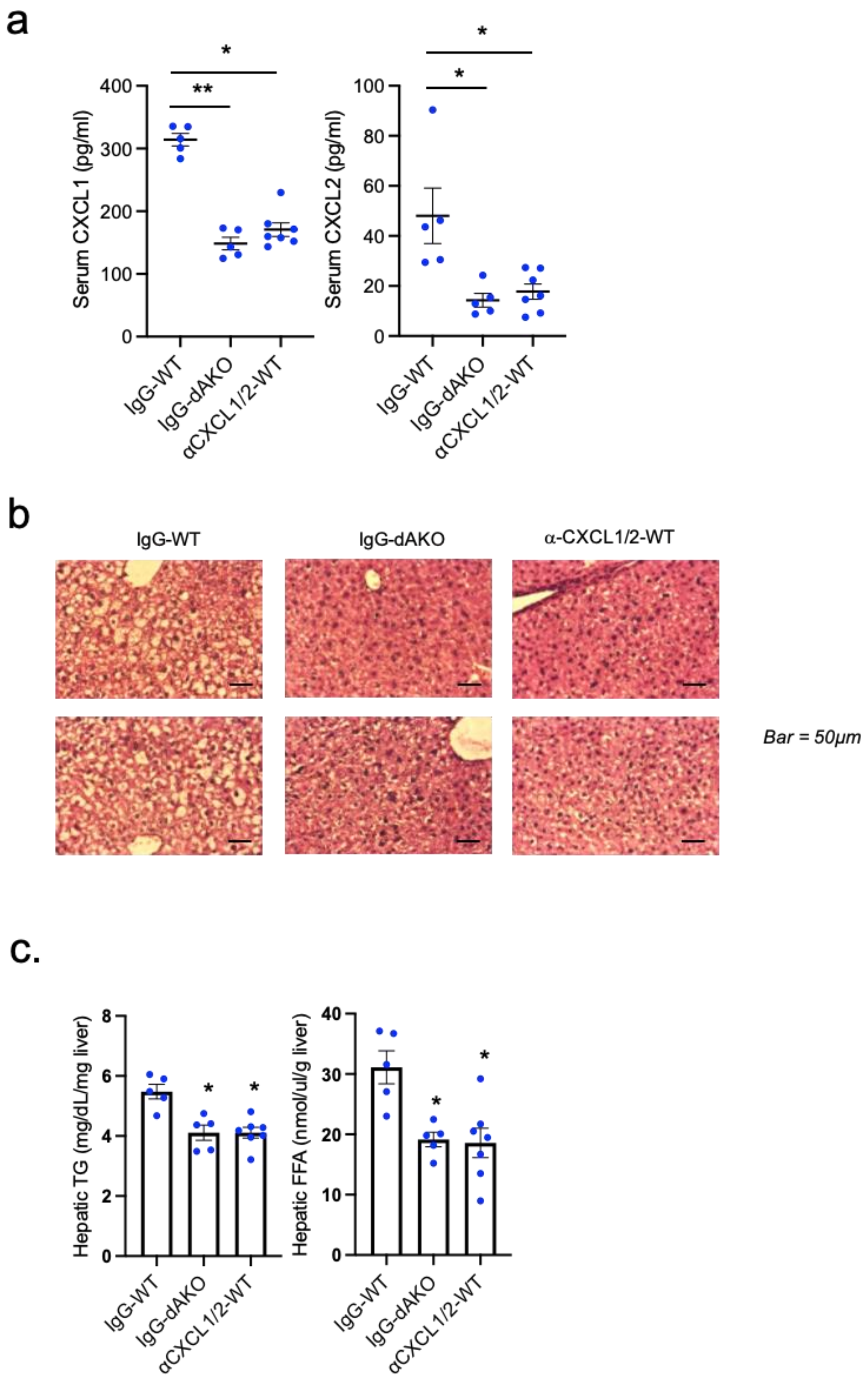

Supplementary Fig 7. **Neutralizing antibody against CXCL1 and CXCL2 reduces inflammation and improves lipid metabolism.**

a. Circulating levels of CXCL1 and CXCL2 (CXCL1/2) in IgG-injected WT, IgG-injected dAKO and α-CXCL1/2–injected WT mice (**P<0.01, *P<0.05, one-way ANOVA; n=5-7). Mice were fed an HFD for 9 weeks. **b.** H&E staining performed on liver sections from IgG-injected WT, IgG-injected dAKO and α-CXCL1/2 injected WT mice. Representative images from 5 mice are shown. **c.** Hepatic triglycerides (TG) and free fatty acids (FFA) in IgG-injected WT, IgG-injected dAKO and α-CXCL1/2-injected WT mice (**P<0.01, *P<0.05, one-way ANOVA; n=5~7).

d

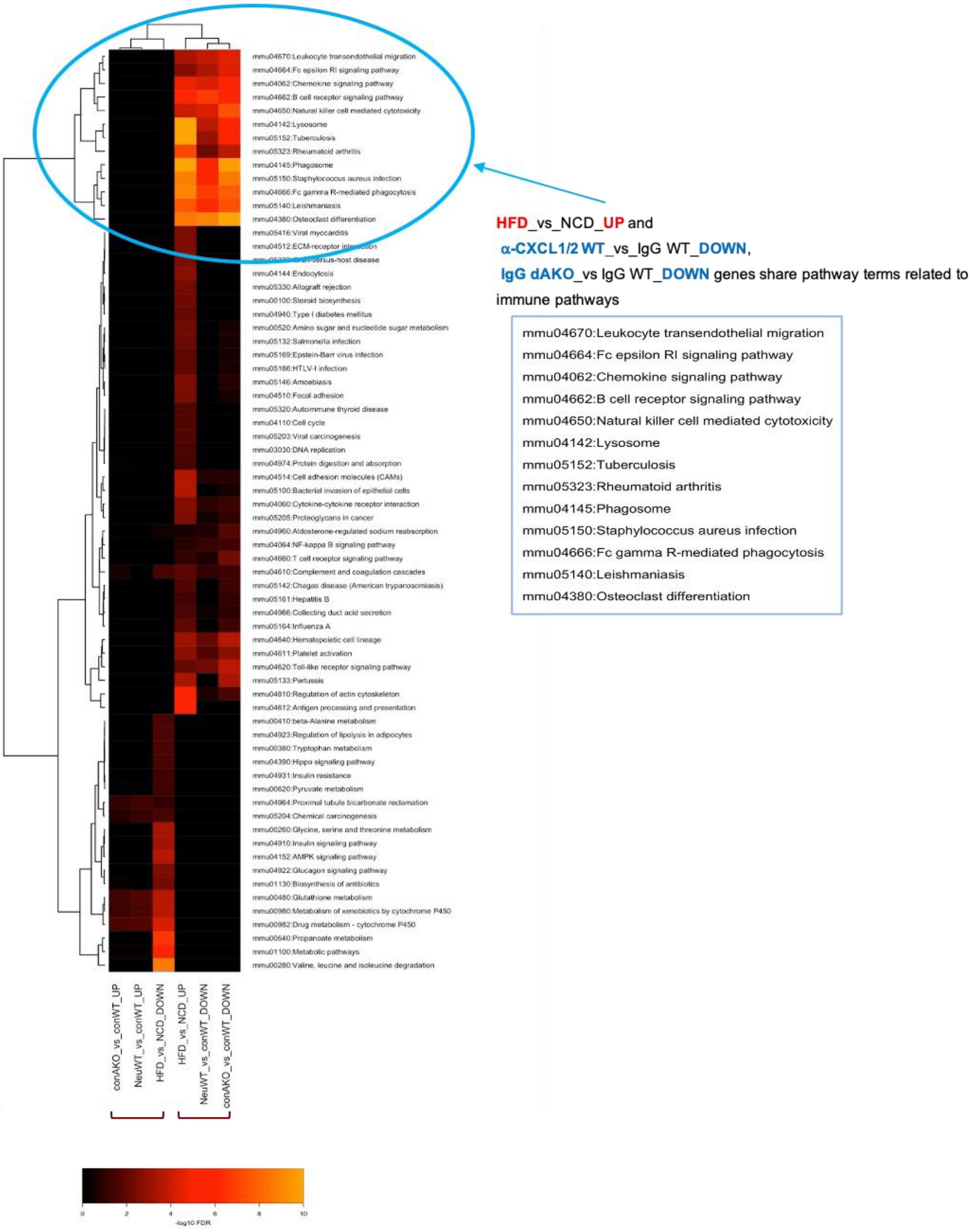

(continued)

d. Heat map from gene expression profile and gene ontology analysis of IgG-WT, IgG-dAKO and α-CXCL1/2-WT mice. Heat map generated from gene expression profile and gene ontology analysis of Fig.1 and Fig.5. Genes that differ more than 2-fold (log FC >1) with a q value (adjusted p-value) less than 0.05 (n=3 per group) shown. Data in **a** and **c** represent the mean ± SEM.

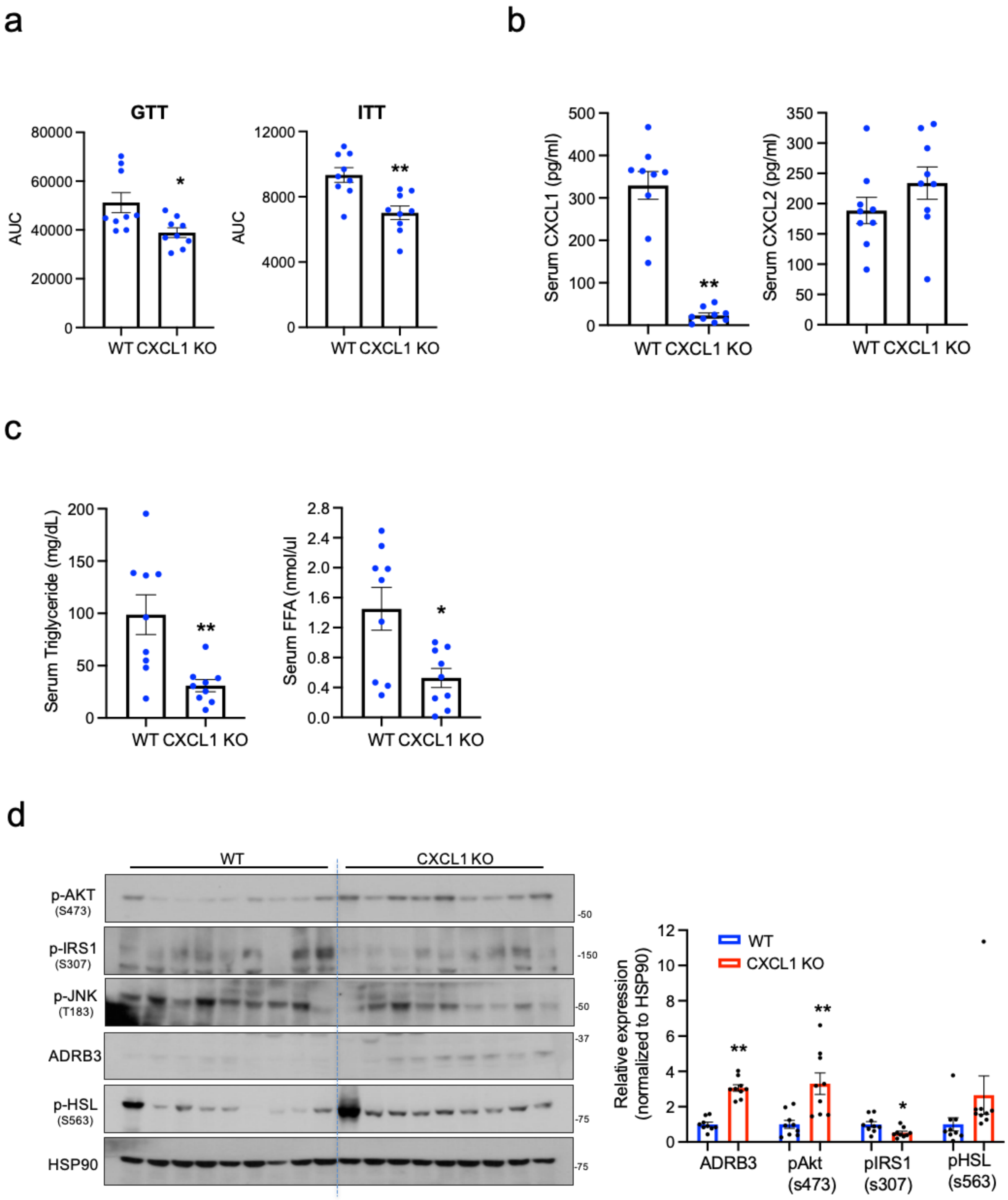

Supplementary Fig 8. **Improved triglyceride metabolism in CXCL1 knockout mice.**
a. Area under curve (AUC) for Fig.6b (**P<0.01, *P<0.05, t-test; n=9 per group). **b.** Circulating CXCL1 and CXCL2 in CXCL1 KO mice and WT littermates after 10 weeks of HFD feeding (**P<0.01, t-test; n=9 per group). **c.** Circulating triglyceride (TG) and free fatty acid (FFA) concentrations in serum from CXCL1 KO mice and WT littermates after 10 weeks of HFD feeding (**P<0.01, *P<0.05, t-test; n=9 per group). **d.** Immunoblot analysis of eWAT from HFD-fed CXCL1 KO and WT littermates showing effect of CXCL1 depletion on insulin, stress, and catecholamine signaling pathways. Right, densitometry analysis for immunoblots shown. Data in **a-d** represent the mean ± SEM.

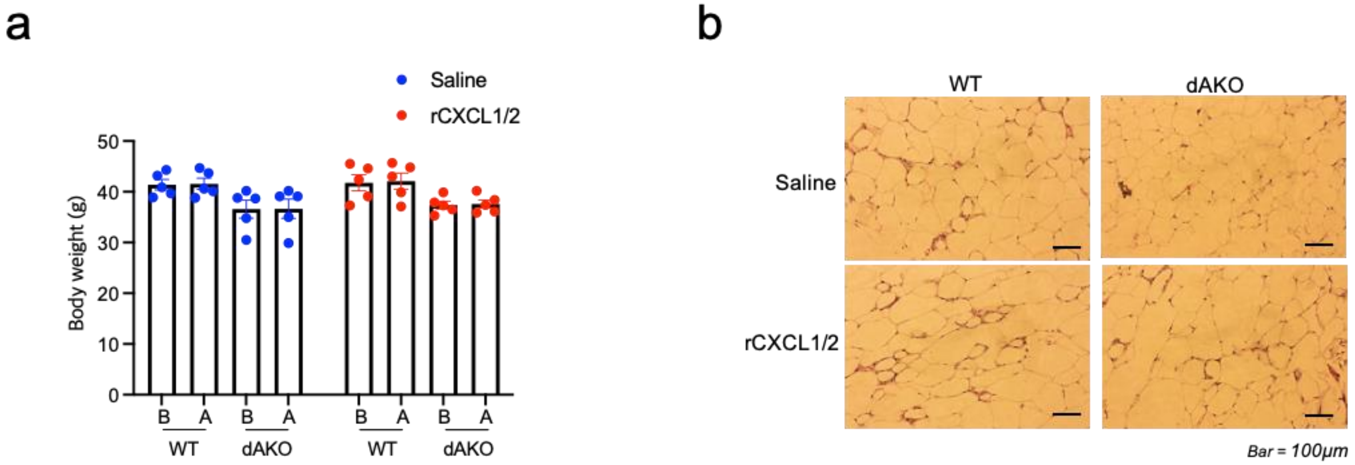

Supplementary Fig 9. **The role of CXCL1/2 on CRTC dAKO mediated regulation.**
a. Body weight of WT and dAKO mice before (B) and after (A) injection of recombinant CXCL1/2 or Saline shown. Data represent the mean ± SEM. **b**. H&E staining of eWAT showing effect of rCXCL1/2 injection in WT and dAKO mice. Representative images shown from 5 mice per group.

a. Uncropped blots for Fig. 1d

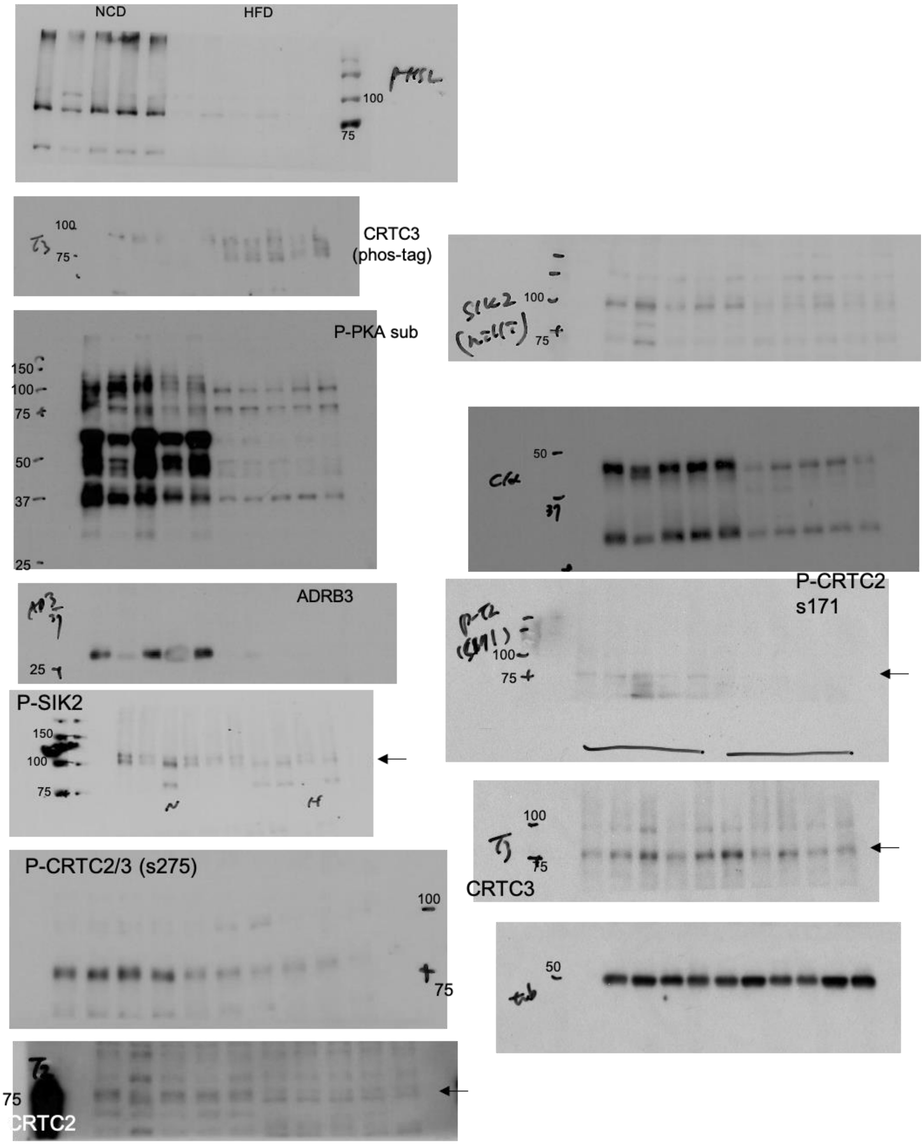

b. Uncropped blots for Fig. 1d

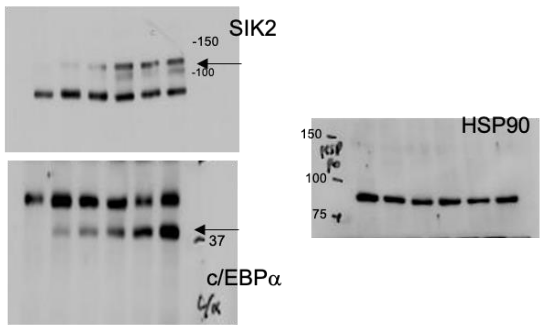

c. Uncropped blots for Fig.2e

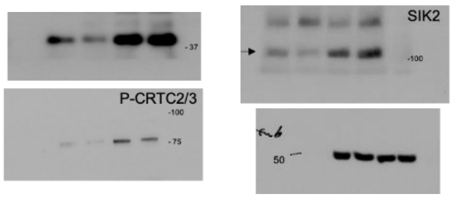

Supplementary Fig 10. Uncropped blots

d. Uncropped blots for Fig. 2f

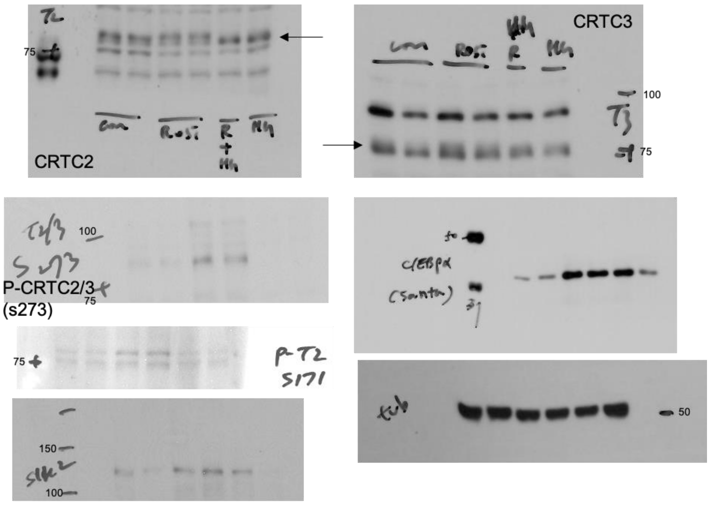

f. Uncropped blots for Fig. 5d

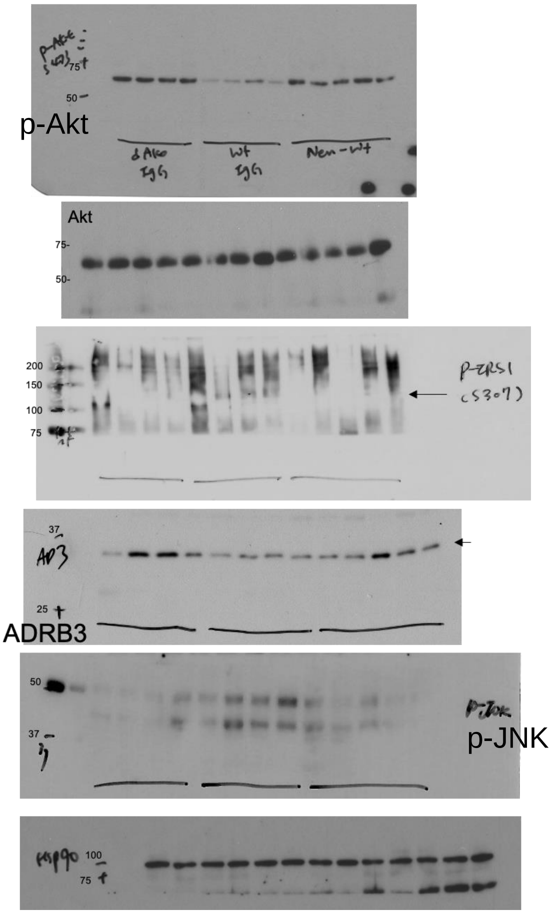

e. Uncropped blots for Fig. 2g

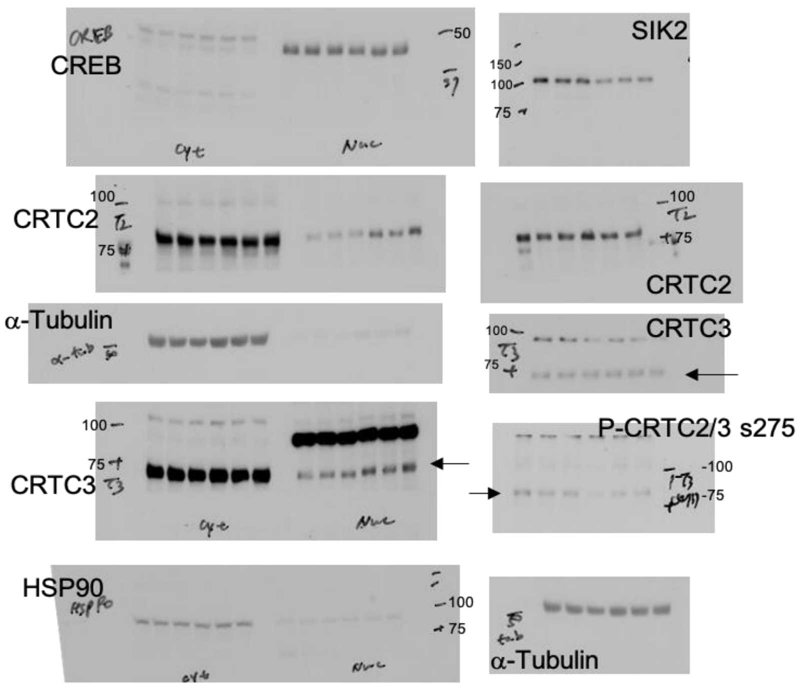

Supplementary Fig 10. Uncropped blots

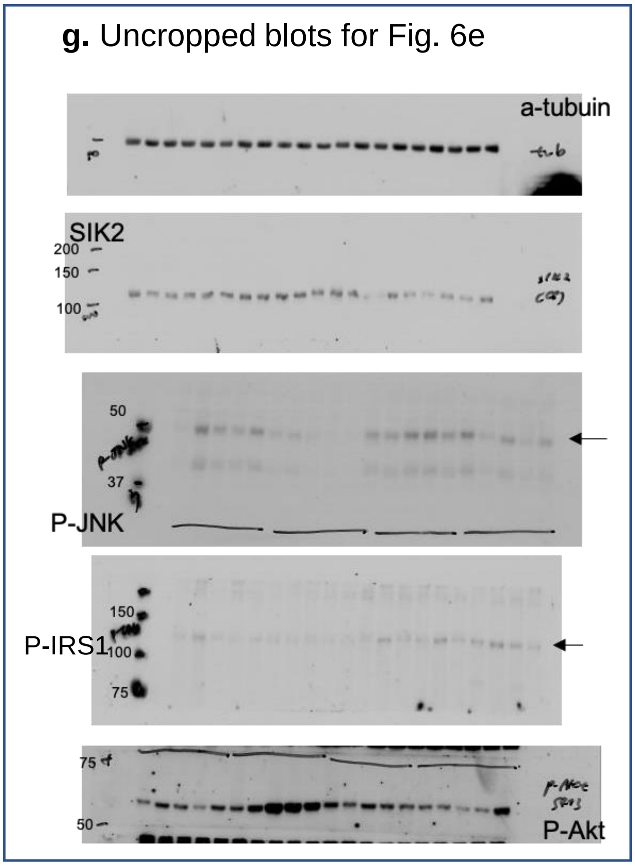

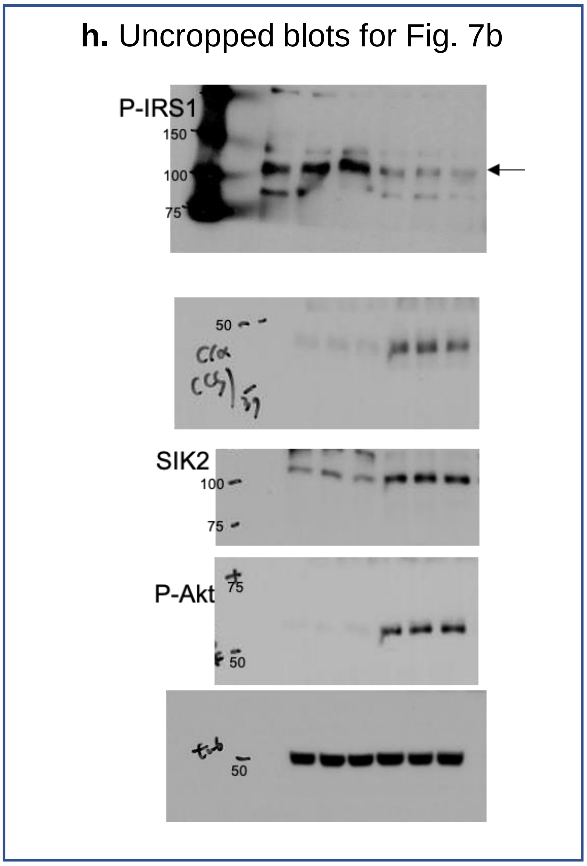

Supplementary Fig 10. Uncropped blots

Supplementary Table 1. All of the genes that are significantly up-regulated upon FSK treatment shown in Supplementary Fig.2

	gene	locus	CON	FSK	log2 FC	p_value	q_value
1	Nr4a2	chr2:57107225-57124003	1.81471	64.0793	5.14205	5.00E-05	0.013718
2	Crem	chr18:3266353-3366863	7.00069	187.006	4.73944	5.00E-05	0.013718
3	Gem	chr4:11704446-11714993	3.65739	78.5121	4.42403	5.00E-05	0.013718
4	Dgat1	chr15:76502014-76511818	8.40065	168.972	4.33014	5.00E-05	0.013718
5	Star	chr8:25808512-25815982	4.6266	88.0137	4.24971	5.00E-05	0.013718
6	Dio2	chr12:90724551-90738438	1.39581	22.9352	4.03838	5.00E-05	0.013718
7	Vdr	chr15:97854426-97908296	17.4382	268.946	3.94699	5.00E-05	0.013718
8	Nfil3	chr13:52967208-52981039	6.19493	78.5003	3.66354	5.00E-05	0.013718
9	Tnfaip6	chr2:52038112-52056681	6.30162	78.7458	3.64341	5.00E-05	0.013718
10	Uap1	chr1:170142002-170174946	55.0574	615.909	3.48371	5.00E-05	0.013718
11	Kcne4	chr1:78816948-78820025	20.9258	232.512	3.47395	5.00E-05	0.013718
12	Slc25a25	chr2:32414486-32455476	3.29954	36.0958	3.45149	5.00E-05	0.013718
13	Tuba4a	chr1:75210828-75219253	11.2557	119.173	3.40432	5.00E-05	0.013718
14	Pde4d	chr13:108654176-109955969	4.26573	44.9586	3.39773	5.00E-05	0.013718
15	Procr	chr2:155751216-155755478	13.588	136.507	3.32856	5.00E-05	0.013718
16	Gfpt2	chr11:49794154-49838620	28.6251	286.436	3.32286	5.00E-05	0.013718
17	Cxcl1	chr5:90891244-90893115	13.6161	121.119	3.15304	5.00E-05	0.013718
18	Ier3	chr17:35821712-35822911	37.4684	319.875	3.09376	5.00E-05	0.013718
19	Nmrk1	chr19:18632015-18652184	6.00127	49.594	3.04683	0.0002	0.041562
20	Adamts4	chr1:171250421-171259922	4.94603	39.3649	2.99257	5.00E-05	0.013718
21	Adamts9	chr6:92772698-92901441	3.07958	23.843	2.95276	5.00E-05	0.013718
22	Sik1	chr17:31844249-31855792	2.30822	17.7126	2.93992	5.00E-05	0.013718
23	Lif	chr11:4257567-4272514	5.54307	41.4847	2.90382	5.00E-05	0.013718
24	Gpr153	chr4:152274361-152285337	3.89162	28.7483	2.88503	5.00E-05	0.013718
25	Fgl2	chr5:21292960-21424677	2.79536	18.8952	2.75691	0.0001	0.02411
26	Jarid2	chr13:44730773-44921643	3.07193	19.8347	2.69081	5.00E-05	0.013718
27	Arrdc3	chr13:80883421-80896043	7.50407	45.8465	2.61107	5.00E-05	0.013718
28	Plau	chr14:20836661-20843388	28.5974	173.219	2.59864	5.00E-05	0.013718
29	Slc16a1	chr3:104638667-104658462	10.5667	63.0647	2.57731	5.00E-05	0.013718
30	Il6	chr5:30013160-30019968	169.221	947.157	2.48469	5.00E-05	0.013718
31	Emd	chrX:74254838-74257747	50.8308	262.959	2.37107	5.00E-05	0.013718
32	Ugdh	chr5:65413221-65435842	163.999	842.094	2.36029	5.00E-05	0.013718
33	Il11	chr7:4772377-4778141	14.6416	73.6977	2.33155	5.00E-05	0.013718
34	Spsb1	chr4:149896283-149955006	6.69612	33.047	2.30312	0.0001	0.02411
35	Synpo2	chr3:123076518-123236149	2.19516	10.7255	2.28865	0.00025	0.047682
36	Ndrg1	chr15:66929317-66969641	11.3652	55.1332	2.2783	0.0001	0.02411
37	Rpl41	chr10:128548109-128549168	152.067	735.95	2.2749	5.00E-05	0.013718
38	Isg15	chr4:156199423-156200818	36.0359	172.968	2.26299	0.00015	0.032633
39	Smim3	chr18:60474190-60501983	13.7557	64.8186	2.23638	0.0002	0.041562
40	Cxcr7	chr1:90203979-90215722	12.7857	59.5117	2.21864	5.00E-05	0.013718
41	Thbd	chr2:148404470-148408188	73.4222	341.357	2.21699	5.00E-05	0.013718
42	Gadd45a	chr6:67035095-67080652	27.741	128.938	2.21658	5.00E-05	0.013718
43	Isy1	chr6:87818447-87838759	13.7804	63.85	2.21207	0.00025	0.047682
44	Ubb	chr11:62551503-62553212	66.4351	268.837	2.01672	5.00E-05	0.013718
45	Tubb2a	chr13:34074279-34078008	32.7727	126.716	1.95104	0.00025	0.047682
46	Bag3	chr7:128523582-128546979	35.2131	132.612	1.91303	0.00015	0.032633
47	Efemp2	chr19:5474689-5481854	80.1825	259.859	1.69637	0.0002	0.041562

Fig 3a

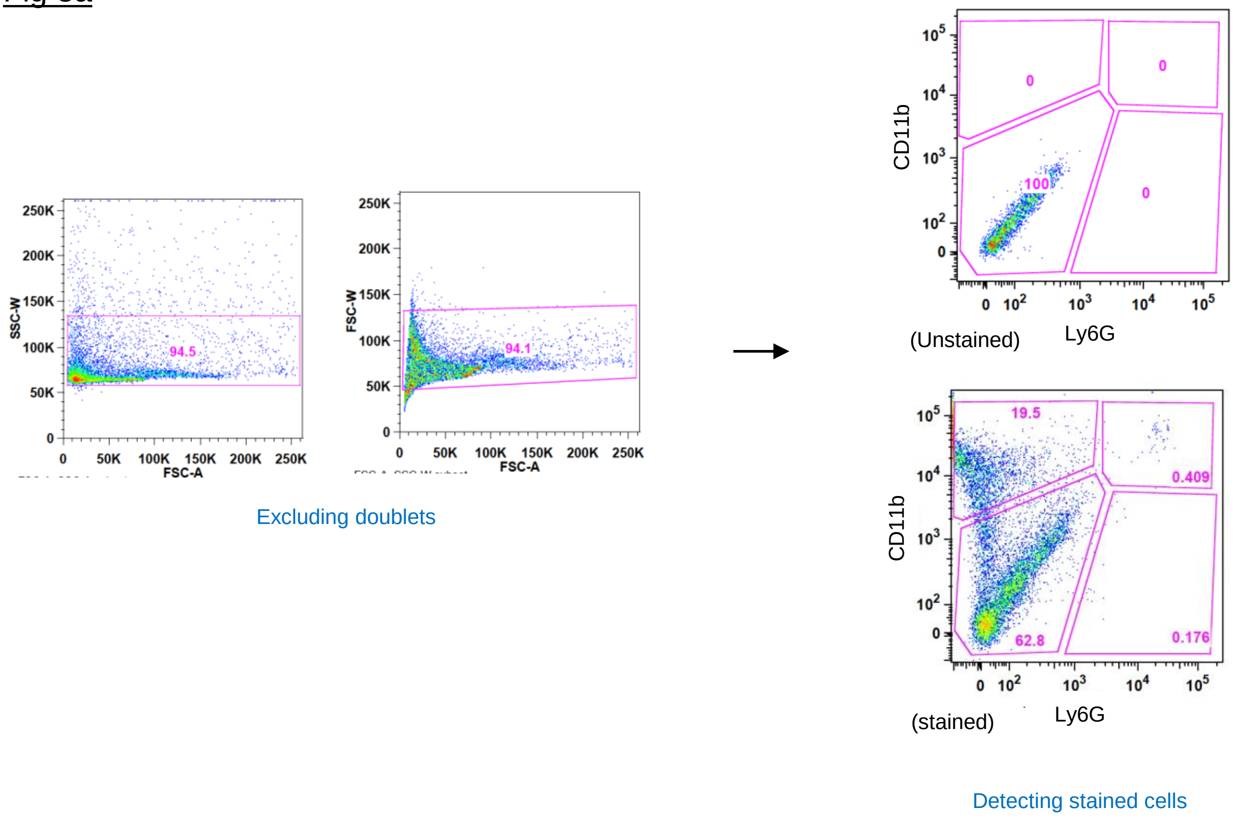

Fig 4f

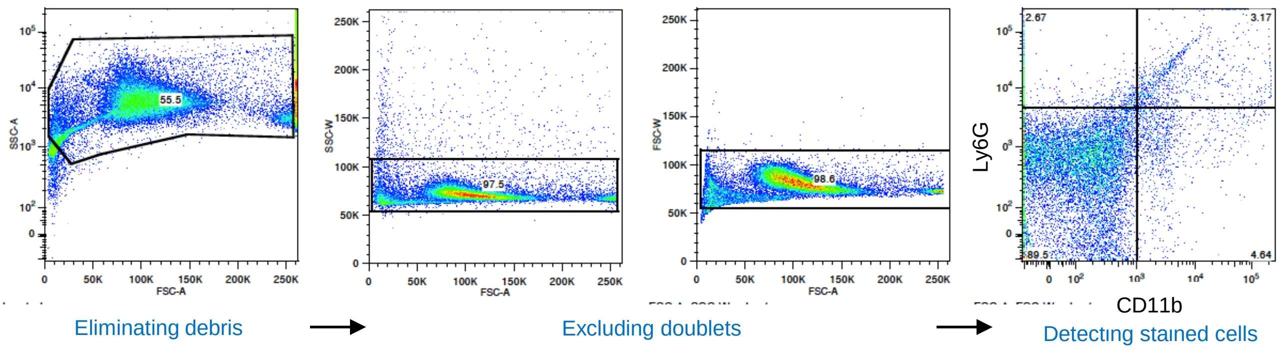

Supplemental item1. Representative gating strategy for neutrophils CD11b+;Ly6g+ (in Fig3a, 4f)

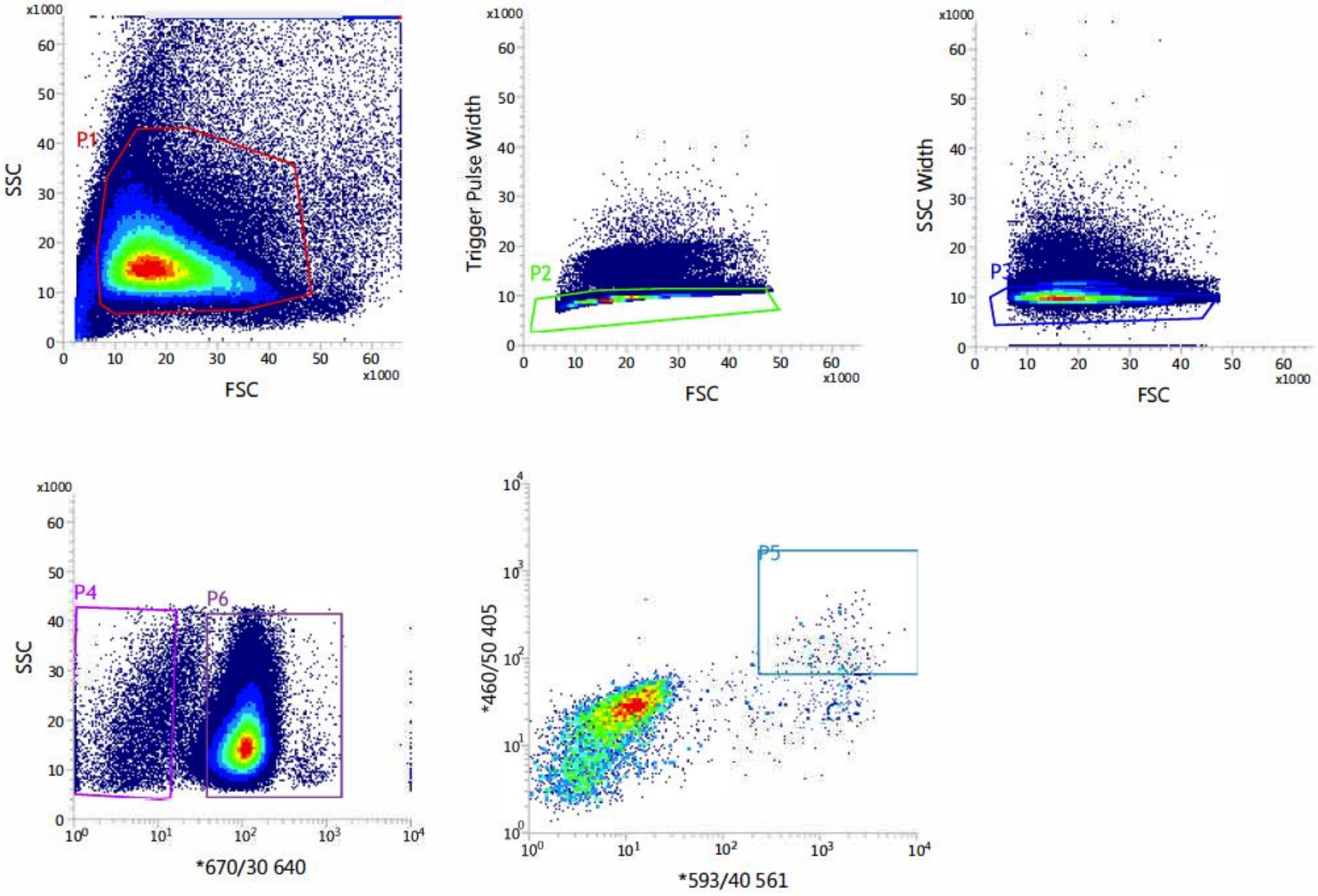

Populations: NC

Populations	Even...	% Total	% Parent	
All Events	392,866	100.00%	####	
P1	365,511	93.04%	93.04%	-Live cells
P2	351,101	89.37%	96.06%	excluding doublets
P3	310,186	78.95%	88.35%	
P4	5,069	1.29%	1.63%	-unstained cells or lineage negative cells
P5	154	0.04%	3.04%	-lineage negative cells
P6	300,753	76.55%	96.96%	-APC CD31; CD45; Ter119 positive cells

Supplemental item2. Representative gating strategy for lineage positive cells (in Supplemantary Fig 1e)
