# Supplementary material for: Activation of the adipocyte CREB/CRTC pathway in obesity
Source: Commun Biol. 2021 Oct 22;4:1214. doi: 10.1038/s42003-021-02735-5 (PMC8536733; doi:10.1038/s42003-021-02735-5)
Supplement: Supplementary file 2 — Description of Additional Supplementary Files [file 42003_2021_2735_MOESM2_ESM.pdf]

# Description of Additional Supplementary Files

**File name:** Supplementary Data.
**Description:** Source Data for figures 1c-7c.
