# Supplementary material for: Activation of the adipocyte CREB/CRTC pathway in obesity
Source: Commun Biol. 2021 Oct 22;4:1214. doi: 10.1038/s42003-021-02735-5 (PMC8536733; doi:10.1038/s42003-021-02735-5)
Supplement: Supplementary file 4 — Reporting Summary [file 42003_2021_2735_MOESM4_ESM.pdf]

nature research

Corresponding author(s): Marc Montminy
Last updated by author(s): Sep 3, 2021

Reporting Summary

Nature Research wishes to improve the reproducibility of the work that we publish. This form provides structure for consistency and transparency in reporting. For further information on Nature Research policies, see our Editorial Policies and the Editorial Policy Checklist.

Statistics

For all statistical analyses, confirm that the following items are present in the figure legend, table legend, main text, or Methods section.

n/a	Confirmed	
☐	☒	The exact sample size (*n*) for each experimental group/condition, given as a discrete number and unit of measurement
☐	☒	A statement on whether measurements were taken from distinct samples or whether the same sample was measured repeatedly
☐	☒	The statistical test(s) used AND whether they are one- or two-sided<br>*Only common tests should be described solely by name; describe more complex techniques in the Methods section.*
☒	☐	A description of all covariates tested
☒	☐	A description of any assumptions or corrections, such as tests of normality and adjustment for multiple comparisons
☐	☒	A full description of the statistical parameters including central tendency (e.g. means) or other basic estimates (e.g. regression coefficient) AND variation (e.g. standard deviation) or associated estimates of uncertainty (e.g. confidence intervals)
☐	☒	For null hypothesis testing, the test statistic (e.g. *F*, *t*, *r*) with confidence intervals, effect sizes, degrees of freedom and *P* value noted<br>*Give P values as exact values whenever suitable.*
☒	☐	For Bayesian analysis, information on the choice of priors and Markov chain Monte Carlo settings
☒	☐	For hierarchical and complex designs, identification of the appropriate level for tests and full reporting of outcomes
☒	☐	Estimates of effect sizes (e.g. Cohen's *d*, Pearson's *r*), indicating how they were calculated

Our web collection on statistics for biologists contains articles on many of the points above.

Software and code

Policy information about availability of computer code

Data collection	All software is commercially or freely available and described in manuscript.
Data analysis	All software is commercially or freely available and described in manuscript. The data were analyzed using Microsoft Excel, Graphpad Prism8.

For manuscripts utilizing custom algorithms or software that are central to the research but not yet described in published literature, software must be made available to editors and reviewers. We strongly encourage code deposition in a community repository (e.g. GitHub). See the Nature Research guidelines for submitting code & software for further information.

Data

Policy information about availability of data

All manuscripts must include a data availability statement. This statement should provide the following information, where applicable:

- Accession codes, unique identifiers, or web links for publicly available datasets
- A list of figures that have associated raw data
- A description of any restrictions on data availability

RNA sequencing and ChIP sequencing data described in this manuscript are deposited in the NCBI Gene Expression Omnibus (GSE160684 for RNA seq and GSE160597 for ChIP seq). Accession Tocken codes for reviewer are ulixqyuszrwjvap (RNA seq) and oxmpkqkotfidbuz (ChIP seq).

Field-specific reporting

Please select the one below that is the best fit for your research. If you are not sure, read the appropriate sections before making your selection.

☒ Life sciences ☐ Behavioural & social sciences ☐ Ecological, evolutionary & environmental sciences

For a reference copy of the document with all sections, see nature.com/documents/nr-reporting-summary-flat.pdf

Life sciences study design

All studies must disclose on these points even when the disclosure is negative.

Sample size	Most experiments are done in triplicates except for animal studies, which involved at least 5 mice per experimental group (age and sex matched).
Data exclusions	No data were excluded.
Replication	All attempts at replication reliably supported conclusions described in the manuscript.
Randomization	Samples or animal were randomly allocated to each group without bias.
Blinding	This is not relevant to this study, since no subjective rating of data was involved.

Reporting for specific materials, systems and methods

We require information from authors about some types of materials, experimental systems and methods used in many studies. Here, indicate whether each material, system or method listed is relevant to your study. If you are not sure if a list item applies to your research, read the appropriate section before selecting a response.

Materials & experimental systems

n/a	Involved in the study	
☐	☒	Antibodies
☐	☒	Eukaryotic cell lines
☒	☐	Palaeontology and archaeology
☐	☒	Animals and other organisms
☒	☐	Human research participants
☒	☐	Clinical data
☒	☐	Dual use research of concern

Methods

n/a	Involved in the study	
☐	☒	ChIP-seq
☐	☒	Flow cytometry
☒	☐	MRI-based neuroimaging

Antibodies

Antibodies used	Antisera are described in the manuscript. We used all commercial antibodies as a 1:1000 dilution,except alpha-tubuline and HSP90 (1:3000). We used all home made antibodies as a 1:3000 dilution. Cell Signaling Technologies (CRTC3;#2720 , pPKA substrate; #9624, c/EBPa; #8178, SIK2; #6919, HDAC4; #5392, Glut4; #2213, p-HSL S563; #4139, p-Akt S473; #4060, p-p38 T180/Y182; #4511, p-p65 S536; #3033, Ac-p65 K310; #12629, p-JNK T183/Y185; #4668, p-AMPK T172; #2535), Santa Cruz Biotechnology (c/EBPa; sc-61, ATF3; sc-518032, JunB; sc-8051, HSP90; sc-7947), Millipore (SIK2; 07-1378, a-tubulin; 05-829), LS Bio (ADRB3; LS-C120555), Boster Bio (CD11b; PB9140), Invitrogen (p-IRS1 S307; PA1-1054, p-SIK2 Thr175; PA5-64607), ABclonal (PTGER3; A4057) and homemade (CRTC2, p-CRTC2 S171, p-CRTC2/3 S275/273).
Validation	All commercial antibodies validated by manufacturer using relevant stress/stimulus and cited in more than 3 publications. Home-made phospho-specific antibodies validated with stimulus. Knockout tissue and cell line samples als used to validate in house antisera.

Eukaryotic cell lines

Policy information about cell lines

Cell line source(s)	All cell lines (293T, 3T3L1, HIB1b, Raw264.7) used in this study are from ATCC.
Authentication	We did not authenticate the 293T cells. We authenticated 3T3L1 and HIB1b cells by checking for induction of adipogenic factors after adipocyte differentiation in vitro.
Mycoplasma contamination	Cell lines used in this study were not contaminated with mycoplasma (Lonza; LT07-118).

Commonly misidentified lines (See ICLAC register)	None of the cell lines we used is listed in ICLAC database.

Animals and other organisms

Policy information about studies involving animals; ARRIVE guidelines recommended for reporting animal research

Laboratory animals	We used C57BL/6 strain of mice. We used male CRTC2/3 double floxed mice with or without adiponectin-Cre recombinase. Age range is 11-18 weeks.
Wild animals	No wild animals are used in our study.
Field-collected samples	No field-collected samples were involved in our study.
Ethics oversight	All procedures involving the use of animals were performed in accordance with the guidelines presented by Salk Institute's Animal Care and Use Committee.

Note that full information on the approval of the study protocol must also be provided in the manuscript.

ChIP-seq

Data deposition

☒ Confirm that both raw and final processed data have been deposited in a public database such as GEO.

☐ Confirm that you have deposited or provided access to graph files (e.g. BED files) for the called peaks.

Data access links *May remain private before publication.*	https://www.ncbi.nlm.nih.gov/geo/query/acc.cgi?acc=GSE160597<br><br>Secure token<br><br>oxmpkqkotfidbuz
Files in database submission	GSM4875778<br>3T3L1-CRTC2-control<br>GSM4875779<br>3T3L1-CRTC2-FSK<br>GSM4875780<br>3T3L1-CRTC2-TNFa<br>GSM4875781<br>3T3L1-CRTC2-FSKTNFa<br>GSM4875782<br>3T3L1-p65-control<br>GSM4875783<br>3T3L1-p65-FSK<br>GSM4875784<br>3T3L1-p65-TNFa<br>GSM4875785<br>3T3L1-p65-FSKTNFa<br>GSM4875786<br>3T3L1-H3AcK27-control<br>GSM4875787<br>3T3L1-H3AcK27-FSK<br>GSM4875788<br>3T3L1-H3AcK27-TNFa<br>GSM4875789<br>3T3L1-H3AcK27-FSKTNFa<br>GSM4875790<br>input-control<br>GSM4875791<br>input-FSK
Genome browser session (e.g. UCSC)	None

Methodology

Replicates	None
Sequencing depth	Sample Read number Uniquely mapped reads Average read length (nt) Single/double end<br>CRTC2-control 18885214 7811598 50.764 single<br>CRTC2-FSK 16438040 9303759 50.760 single

CRTC2-TNFa 26472345 9414862 50.784 single
CRTC2-FSKTNFa 31807394 13026109 50.782 single
p65-control 26505421 18504780 50.777 single
p65-FSK 24941629 4559667 5 0.769 single
p65-TNFa 26904732 6106981 50.772 single
p65-FSKTNFa 20490261 4891561 50.765 single
H3AcK27-control 27601287 20524212 50.798 single
H3AcK27-FSK 9832884 8044186 50.730 single
H3AcK27-TNFa 26904732 6106981 50.772 single
H3AcK27-FSKTNFa 25729060 18273689 50.786 single
input-control 25700078 19561531 50.768 single
input-FSK 27193734 17643239 50.768 single

Antibodies

CRTC2 in house serum #6865

p65 Cell Signaling #8242

H3AcK27 AbCam Ab4729

Peak calling parameters

Reads were aligned to the mm10 genome build using STAR.

Peaks were called with Homer using the findPeaks program against input (no IP) DNA files. For CRTC2 and p65, the "factor" mode was used e.g.:

findPeaks CRTC23T3FSK_tagdir/ -style factor -o CRTC2FSKpeaks -i input3T3FSK_tagdir/

For H3AcK27, the "histone" mode was used e.g.:

findPeaks HAcK3T3FSK_tagdir/ -style histone -o HAcKFSKpeaks -i input3T3FSK_tagdir

Data quality

Peak quality was calculated using Homer (findPeaks) with default parameters: FDR = 0.001, tag density in peaks requires four fold greater than in the surrounding 10kb region and four fold greater than input.

p65 immunoprecipitation was not efficient (0.63% based on tags found in peaks versus background for FSK+TNFa treatment), but NFkB binding motif was clearly enriched under p65 peaks.

Number of peaks that passed thresholds:

Sample Peak number
CRTC2-control 5188
CRTC2-FSK 11749
CRTC2-TNFa 19527
CRTC2-FSKTNFa 14171
p65-control 214
p65-FSK 2969
p65-TNFa 2404
p65-FSKTNFa 4221
H3AcK27-control 52321
H3AcK27-FSK 38684
H3AcK27-TNFa 49754
H3AcK27-FSKTNFa 42227

Software

STAR for alignment of reads to the mm10 mouse genome build.

HOMER for peak calling, quantification, annotation and visualization on the UCSC genome browser.

Flow Cytometry

Plots

Confirm that:

☒ The axis labels state the marker and fluorochrome used (e.g. CD4-FITC).

☒ The axis scales are clearly visible. Include numbers along axes only for bottom left plot of group (a 'group' is an analysis of identical markers).

☒ All plots are contour plots with outliers or pseudocolor plots.

☒ A numerical value for number of cells or percentage (with statistics) is provided.

Methodology

Sample preparation	primary stromal vascular fraction (SVF) cells were isolated from mouse epidydimal adipose tissue. Following isolation, SVF cells were labeled with fluorochrome tagged markers and then washed (HBSS +2% FBS) and analyzed.
Instrument	BD FACS LSRII(BD Biosciences)
Software	The cytometer ran BD FACSDiva acquisition software and FlowJo v9.7.5.
Cell population abundance	Cell sorting was not employed
Gating strategy	Using the Initial FSC-A/SSC-A gating, cell debris was excluded by gating on the main cell population. Then cells were gated in FSC-W/FSC-A to discriminate single cells; then cells were gated in SSC-W/SSC-A to discriminate live cells. Positive threshold for each marker was defined on WT cells in control group and then identical positive threshold was applied to other cells in test group. Un-labelled cells were used for selecting negative population.

☒ Tick this box to confirm that a figure exemplifying the gating strategy is provided in the Supplementary Information.
